# Conserved NIMA kinases regulate multiple steps of endocytic trafficking

**DOI:** 10.1371/journal.pgen.1010741

**Published:** 2023-04-26

**Authors:** Braveen B. Joseph, Naava Naslavsky, Shaonil Binti, Sylvia Conquest, Lexi Robison, Ge Bai, Rafael O. Homer, Barth D. Grant, Steve Caplan, David S. Fay

**Affiliations:** 1 Department of Molecular Biology, College of Agriculture Life Sciences, and Natural Resources, University of Wyoming, Laramie, Wyoming, United States of America; 2 Department of Biochemistry & Molecular Biology, University of Nebraska Medical Center, Omaha, Nebraska, United States of America; 3 The Fred and Pamela Buffett Cancer Center, University of Nebraska Medical Center, Omaha, Nebraska, United States of America; 4 Department of Molecular Biology and Biochemistry, Rutgers University, Piscataway, New Jersey, United States of America; University of California San Diego, UNITED STATES

## Abstract

Human NIMA-related kinases have primarily been studied for their roles in cell cycle progression (NEK1/2/6/7/9), checkpoint–DNA-damage control (NEK1/2/4/5/10/11), and ciliogenesis (NEK1/4/8). We previously showed that *Caenorhabditis elegans* NEKL-2 (NEK8/9 homolog) and NEKL-3 (NEK6/7 homolog) regulate apical clathrin-mediated endocytosis (CME) in the worm epidermis and are essential for molting. Here we show that NEKL-2 and NEKL-3 also have distinct roles in controlling endosome function and morphology. Specifically, loss of NEKL-2 led to enlarged early endosomes with long tubular extensions but showed minimal effects on other compartments. In contrast, NEKL-3 depletion caused pronounced defects in early, late, and recycling endosomes. Consistently, NEKL-2 was strongly localized to early endosomes, whereas NEKL-3 was localized to multiple endosomal compartments. Loss of NEKLs also led to variable defects in the recycling of two resident cargoes of the trans-Golgi network (TGN), MIG-14/Wntless and TGN-38/TGN38, which were missorted to lysosomes after NEKL depletion. In addition, defects were observed in the uptake of clathrin-dependent (SMA-6/Type I BMP receptor) and independent cargoes (DAF-4/Type II BMP receptor) from the basolateral surface of epidermal cells after NEKL-2 or NEKL-3 depletion. Complementary studies in human cell lines further showed that siRNA knockdown of the NEKL-3 orthologs NEK6 and NEK7 led to missorting of the mannose 6-phosphate receptor from endosomes. Moreover, in multiple human cell types, depletion of NEK6 or NEK7 disrupted both early and recycling endosomal compartments, including the presence of excess tubulation within recycling endosomes, a defect also observed after NEKL-3 depletion in worms. Thus, NIMA family kinases carry out multiple functions during endocytosis in both worms and humans, consistent with our previous observation that human NEKL-3 orthologs can rescue molting and trafficking defects in *C*. *elegans nekl-3* mutants. Our findings suggest that trafficking defects could underlie some of the proposed roles for NEK kinases in human disease.

## Author summary

Intracellular trafficking is an evolutionary conserved process whereby cargoes, which include proteins, lipids, and other macromolecules, are internalized by cells, packaged into vesicles, and distributed to their proper places within the cell. This study demonstrated that two conserved NIMA-related kinases, NEKL-2 and NEKL-3, are required for the transport of multiple cargoes in the epidermis of *C*. *elegans*. NEKL-2 and NEKL-3 function at organelles, called endosomes, to regulate their morphology and control the sorting of cargoes between different intracellular compartments. In the absence of NEKL activities, various cargoes, including components of the BMP and Wnt signaling pathways, were misregulated. Our studies are further supported by results showing that the human counterparts of NEKL-3, NEK6 and NEK7, were also required for maintaining endosome morphologies and for the proper sorting of cargo in human cells. Notably, NIMA-kinases are well studied for their roles in cell cycle regulation, and overexpression of these kinases is linked to cancer formation and poor prognosis. Our study suggests their role in cancer progression could be partly due to the abnormal intracellular trafficking of conserved signaling components with known roles in cancer formation.

## Introduction

The NIMA (never in mitosis gene a) family of serine/threonine kinases was first identified in a genetic screen for mutants that fail to enter mitosis in the filamentous fungus *Aspergillus nidulans* [1–4]. The human genome encodes 11 NIMA-related kinases or NEKs (NEK1–NEK11). Consistent with the pro-mitotic function of NIMA in *A*. *nidulans*, several mammalian NEKs regulate cell division processes including spindle assembly and centrosome separation (NEK1, NEK2, NEK5–NEK7, and NEK9) and cytokinesis (NEK1, NEK2, NEK6, and NEK7) [5–16]. Correspondingly, misregulation of NEKs can cause aberrant cell proliferation, and overexpression of several NEKs, including NEK6, NEK7, and NEK9, is associated with multiple cancers and cardiac hypertrophy [8,17–20]. More recently, attention on NEK7 has focused on its role as an activator of NLRP3-inflammasomes, which are critical for the immune response against microbial pathogens [21,22]. Other NEK family members (e.g., NEK1, NEK4, and NEK8) were identified as causal factors in mouse models of polycystic kidney disease or have been linked to human ciliopathies [23–30]. Several NEK family members, including NEK8 and NEK9, span multiple functional categories, as NEK8 has been linked to cancer and cell cycle control [18,26,31,32] and NEK9 has been implicated in ciliogenesis and ciliopathies [25,33]. These findings suggest that individual NEKs have tissue- or cell type–specific functions, which may affect different developmental and disease-associated processes.

*C*. *elegans* has four NIMA-related kinases, NEKL-1, NEKL-2, NEKL-3, and NEKL-4. NEKL-2, a homolog of human NEK8 and NEK9, contains a highly conserved kinase domain and a short disordered region at the C terminus [34]. NEKL-2 is expressed in a punctate pattern in the large epidermal syncytium, hyp7, where it colocalizes with the conserved ankyrin repeat proteins MLT-2 (ANKS6 homolog) and MLT-4 (INVS homolog). MLT-2 and MLT-4 are required for the proper localization of NEKL-2, and their physical association is conserved in vertebrates [34–36]. NEKL-3, a close homolog of human NEK6 and NEK7, contains a kinase domain only and is also expressed in a punctate pattern within hyp7. NEKL-3 forms a complex with a third conserved ankyrin repeat protein, MLT-3/ANKS3, which is required for the proper localization of NEKL-3 in *C*. *elegans* and NEK7 in vertebrates [37]. We have shown that NEKL-2 and NEKL-3, along with their MLT binding partners, are required for the completion of molting in *C*. *elegans*, as a strong loss of function in any of these proteins leads to molting defects in early larval stages [34,38–40]. Moreover, mutation of the kinase domain of *nekl-2* or *nekl-3* leads to uniform molting defects indicating that the catalytic activity of the NEKLs is essential for the promotion of molting [34,39].

Molting is an essential feature for *C*. *elegans* to develop through its four larval stages and to reach adulthood. During molting, the old cuticle, an apical extracellular matrix (aECM) made up of proteins and lipids, detaches from the underlying epidermis and is broken down as a new cuticle is synthesized underneath. This process of aECM remodeling requires the trafficking of macromolecules required for the synthesis, degradation, and recycling of cuticle components [41,42]. We previously reported that NEKL-2 and NEKL-3 regulate clathrin-mediated endocytosis (CME) at the apical membrane of hyp7. Our findings indicated that loss of NEKL-2 or NEKL-3 leads to a defect in the uncoating of apical clathrin-coated vesicles, thereby preventing the flow of apical cargo through the endocytic pathway. Consistent with this, an apically expressed low-density lipoprotein–like receptor, LRP-1/Megalin, was observed to be trapped at or near the apical membrane in NEKL-depleted animals [34,43]. LRP-1 internalization by hyp7 is essential for molting, as it is required for the uptake of cholesterol, a substrate required for hormonal signaling pathways that coordinate the molting cycle [41,44,45].

The involvement of NEKLs in CME is also strongly supported by our genetic screens, which identified subunits of the clathrin adapter protein complex, AP2, as strong suppressors of both molting and trafficking defects in *nekl-2* and *nekl-3* mutants. Likewise, loss of FCHO-1, an allosteric activator of AP2, alleviates molting and trafficking defects in *nekl* mutants [43]. Our collective data indicate that NEKL-2 and NEKL-3 directly or indirectly promote clathrin uncoating and suggest that AP2 activity can also affect the uncoating process. However, a possible role for NEKLs in other aspects of intracellular trafficking was not explored.

In this study, we further investigated the role of NEKLs at different membranes and in different compartments within the endocytic network. Importantly, we found that NEKL-2 and NEKL-3 have distinct functions within different endosome types and are required for basolateral trafficking and cargo recycling. Importantly, we extended our studies to human cell lines and demonstrated that the human NEKL-3 orthologs, NEK6 and NEK7, also appear to function at multiple points within the endocytic pathway in mammals. These studies provide new insights into the biological roles of NEK/NEKL family members, which may have relevance to their roles in human development and disease.

## Results

### NEKL-2 and NEKL-3 colocalize with endosomal markers

**Fig 1:**
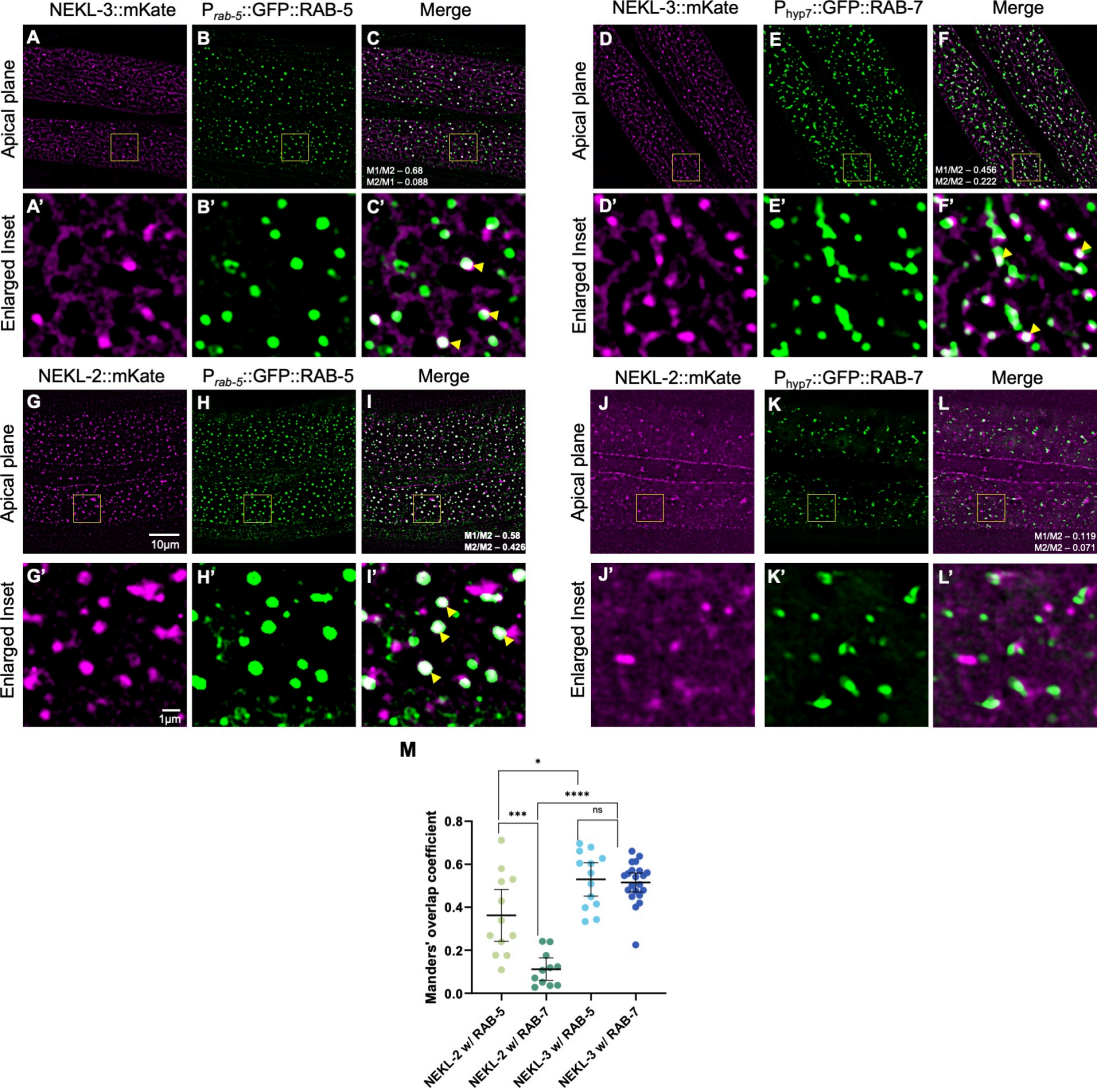
Colocalization of NEKL-2 and NEKL-3 with endosomal markers in *C*. *elegans*. Colocalization assays were carried out in adult worms expressing either NEKL-2::mKate or NEKL-3::mKate with endosomal markers P_*rab-5*_::GFP::RAB-5 or P_hyp7_::GFP::RAB-7. (A–C, A’–C’, G–I, and G’–I’) Representative confocal images of adult worms expressing either NEKL-3::mKate (A–C and A’–C’) or NEKL-2::mKate (G–I and G’–I’), along with the early endosomal marker *P*_*rab-5*_::GFP::RAB-5. (D–F, D’–F’, J–L, and J’–L’) Representative confocal images of adult worms expressing either NEKL-3::mKate (D–F and D’–F’) or NEKL-2::mKate (J–L and J’–L’) and late endosomal marker P_hyp7_::GFP::RAB-7. Yellow arrowheads indicate overlap. Scale bar in A = 10 μm for A–L. Scale bar in A’ = 1 μm for A’–L’. (M) Manders’ coefficient was calculated for all the worms with the indicated backgrounds and plotted to determine the fraction of overlap between NEKL-2– or NEKL-3–positive pixels and the indicated markers. Error bars represent the 95% confidence intervals. p-Values were obtained by comparing means using an unpaired t-test: ****p < 0.0001, ***p < 0.001, *p < 0.05; ns, not significant (p > 0.05). Raw data are available in S1 File.

Previously, we reported that NEKL-2 and NEKL-3 are specifically expressed and required within the large epidermal syncytium of *C*. *elegans*, hyp7 [34,38]. Moreover, both NEKL-2 and NEKL-3 are cytoplasmic and are localized to variably sized actin-rich puncta or aggregates, which are reminiscent of endosomal compartments [34,38]. To determine the subcellular compartments in which NEKLs may function, we carried out colocalization experiments using endogenously tagged NEKL strains, together with reporters for early (P_*rab-5*_::GFP::RAB-5) and late (P_hyp7_::GFP::RAB-7) endosomes [46–50]. In the case of NEKL-3, we observed robust colocalization of NEKL-3::mKate with GFP::RAB-5 and GFP::RAB-7 (~50% Manders’ overlap with each marker), such that a majority of detectable NEKL-3 may be associated with early-to-late endosomes (Fig 1A–1F, 1A’–1F’ and 1M). In contrast, whereas ~40% of NEKL-2::mKate colocalized with RAB-5::GFP, only ~10% colocalized with RAB-7::GFP, suggesting that NEKL-2 may play a greater role in early steps of endocytic trafficking (Fig 1G–1L, 1G’–1L’ and 1M). We also note that we previously reported the association of NEKL-2–MLT-2–MLT-4 with actin-rich puncta that form along the boundary of hyp7 and the seam cells, which do not appear to be endosomal and may represent adhesion junctions [38,39]. In addition, we have observed some diffuse reticulated signal from fluorescently tagged NEKL-3 and NEKL-2 in the cytoplasm of hyp7 (Fig 1), which could correspond in part to the endoplasmic reticulum [39].

The differences observed between NEKL-2 and NEKL-3 localization are consistent with our previous report that NEKL-2 and NEKL-3, along with their MLT co-partners, exhibit distinct localization patterns in hyp7 [39]. Consistent with this, we find that the Manders’ overlap between fluorescently tagged NEKL-2 and NEKL-3 is <20% (S1 Fig), suggesting that the cellular functions of these kinases are distinct, despite having similar terminal molting-defective phenotypes. Notably, although a loss of NEKL-2 and NEKL-3 lead to defects in clathrin uncoating [43], neither showed strong colocalization with a marker for the AP2 clathrin-adapter protein complex, APA-2::mScarlet (S2 Fig). This suggests that the previously described impact of NEKLs on clathrin uncoating may be indirect or that levels of NEKLs at clathrin-coated vesicles are below detection. Notably, both NEKLs colocalize with RAB-5, which plays a role in the uncoating of clathrin-coated vesicles [51].

### NEKLs are required for normal endosome morphologies

Given the observed colocalization of NEKLs with endosomes, we next wanted to determine if the loss of NEKLs led to an observable defect in the morphologies of endosomal compartments. During the process of endocytosis, endosomes undergo maturation, which is accompanied by changes in shape, molecular composition, and function. In the case of early/sorting and recycling endosomes, tubular extensions extend and pinch off from larger vesicles, allowing proteins to be recycled to specific destinations such as the plasma membrane and trans-Golgi [52–55]. Disruptions to endosomal functions can lead to a wide variety of defects including alterations in cargo localization and abundance.

**Fig 2:**
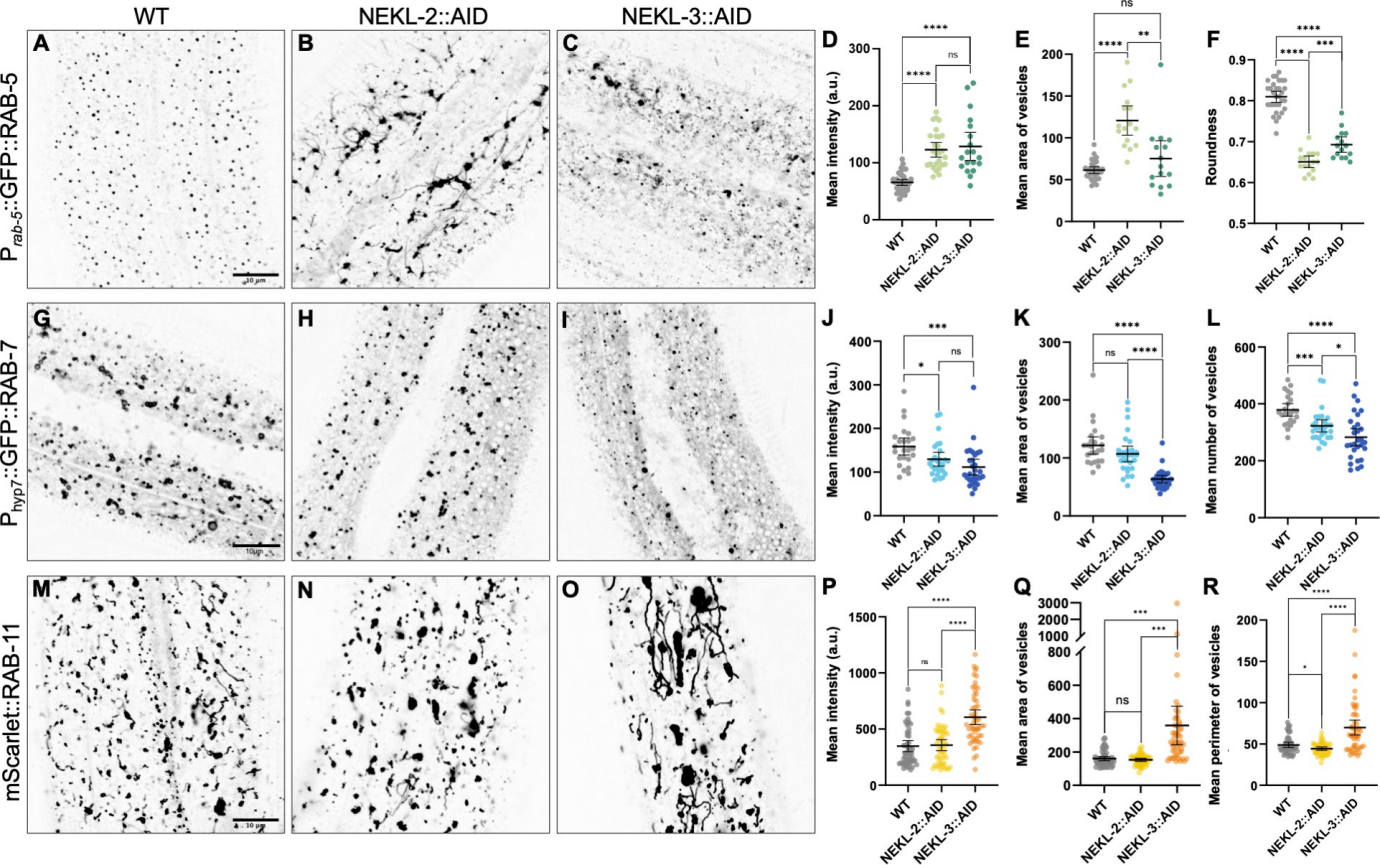
Effects of NEKL-2 or NEKL-3 depletion on endosomal compartments in *C*. *elegans*. (A–C, G–I, M–O) Confocal imaging was used to examine the effects of NEKL-2 (B, H, N) and NEKL-3 (C, I, O) loss relative to wild type (WT; A, G, M) in hyp7 of day-2 adult worms after auxin treatment. Representative images are shown. (A–C) Imaged worms expressed P_*rab-5*_::GFP::RAB-5 in the indicated backgrounds. (D–F) The mean intensity (D), vesicle area (E), and the roundness of the puncta (F) were plotted for worms expressing P_*rab-5*_::GFP::RAB-5. (G–I) Imaged worms expressed P_hyp7_::GFP::RAB-7 in the indicated backgrounds. (J–L) The mean intensity (J), vesicle area (K), and the number of vesicles (L) were plotted for worms expressing P_hyp7_::GFP::RAB-7. (M–O) Imaged worms expressed mScarlet::RAB-11, a marker for recycling endosomes, in the indicated backgrounds. (P–R) The mean intensity (P), vesicle area (Q), and perimeter of the vesicles (R) were plotted for individual worms expressing mScarlet::RAB-11. Area is in square pixels; perimeter is the number of pixels in the boundary of the object. Error bars represent the 95% confidence intervals. p-Values were obtained by comparing means using an unpaired t-test: ****p < 0.0001, ***p < 0.001, *p < 0.05; ns, not significant (p > 0.05). Raw data are available in S1 File.

To avoid indirect consequences caused by molting defects, we assessed the roles of NEKLs on endosomal morphology using the auxin-inducible degron (AID) system, in which endogenously tagged NEKL-2::AID and NEKL-3::AID were depleted in day-1 adults after the addition of auxin [43,56,57]. Depletion of NEKL-2::AID led to an increase in the overall size of the early endosome compartment (based on total intensity and area measurements; P_*rab-5*_::GFP::RAB-5) and, most strikingly, to an increase in the extent of tubulation (Fig 2A, 2B and 2D–2F). This phenotype suggests a requirement for NEKL-2 in the process of vesicle fission at early endosomes. In contrast, loss of NEKL-2 had more modest effects on the late endosome compartment (P_hyp7_::GFP::RAB-7), consistent with the observed strong colocalization of NEKL-2 to early, but not late, endosomes (Fig 2G, 2H and 2J–2L). Specifically, although the mean number of vesicles was decreased in NEKL-2–depleted worms as compared with wild type, the morphology of late endosomes was not strongly affected, and the observed changes could be due in part to gross alterations in the early endosome caused by NEKL-2 depletion.

In the case of NEKL-3::AID, depletion led to strong defects at both early and late endosomes, consistent with the localization of NEKL-3 to both compartments. In the case of early endosomes, however, the effects of NEKL-3 loss differed markedly from those after loss of NEKL-2 (Fig 2B–2F). Rather than increased tubulation, loss of NEKL-3 led to increased variability in the size and shape of early endosomes accompanied by an increase in total signal intensity. Thus, while NEKL-2 and NEKL-3 both act at early endosomes, their roles in controlling early endosome morphology appear to be distinct. Depletion of NEKL-3 also led to a strong overall decrease in the abundance of late endosomes, which was associated with a decrease in total intensity, vesicle size, and changes in vesicle morphology (Fig 2G and 2I–2L). These defects may be due to a combination of the roles for NEKL-3 in directly regulating late endosomes and the potential secondary effects associated with its effects on early endosomes. We also note that even in the absence of auxin, NEKL-3::AID worms are smaller than wild-type worms and show some arrest due to molting defects, which may be attributable to a partial LOF caused by the AID tag [43].

We also examined the effects of NEKL loss on recycling endosomes, which were marked with endogenously (CRISPR) tagged RAB-11. Whereas NEKL-2::AID depletion had little or no effect on mScarlet::RAB-11 localization, we observed a strong increase in tubulation after loss of NEKL-3:AID, which was accompanied by an increase in total intensity and vesicle size (Fig 2M–2R). For technical reasons, we were unable to carry out colocalization studies between the NEKLs and either mScarlet::RAB-11 or GFP::RAB-11. Our observations, however, suggest that NEKL-3 may also function at recycling endosomes, possibly to control vesicle fission. In contrast to endosomal compartments, depletion of NEKL-2::AID and NEKL-3::AID had little or no effect on the morphology of the Golgi compartment, based on a P_hyp7_::AMAN-2::mNeonGreen marker (S3 Fig). Nevertheless, the total number of AMAN-2–positive vesicles was variably reduced in these backgrounds, with strongest effects shown for NEKL-3::AID (S3A–S3C Fig), which could be caused by reduced endosome-to-Golgi recycling. Consistent with an indirect effect, a multi-copy NEKL-3::mCherry reporter [34] showed very low levels of colocalization with AMAN-2:: mNeonGreen (S3D Fig). Collectively, our studies demonstrate a role for NEKL-2 and NEKL-3 in regulating endosomal morphologies and further suggest direct functions within endosomal compartments.

### NEKLs are required for basolateral cargo uptake by hyp7

We previously showed that NEKLs are required for CME at the apical membrane of hyp7 (Fig 1A and 1B) [44,45,58]. Specifically, depletion of NEKL-2 or NEKL-3 led to the accumulation of both clathrin and LRP-1, a low-density lipoprotein–like receptor, at the apical hyp7 surface [34,43,59,60]. Consistent with this, *C*. *elegans* LRP-1 endocytosis is dependent on clathrin and the clathrin adapter protein complex, AP2, analogous to findings in mammals [34,43,60–64]. To determine whether NEKL kinases are also required for trafficking at basolateral membranes of hyp7, we examined the internalization of two basolaterally localized BMP-family receptors. SMA-6 (Type I BMP receptor) and DAF-4 (Type II BMP receptor) form a heteromeric complex that binds to DBL-1 (BMP ligand), leading to the regulation of genes controlling *C*. *elegans* body size and male mating structures [65]. The internalization of SMA-6 depends on CME, as loss of AP2 function leads to the accumulation of SMA-6 at the basolateral membrane of intestinal cells [66]. In contrast, DAF-4 internalization does not require AP2, suggesting that it is endocytosed through a clathrin-independent mechanism [66].

**Fig 3:**
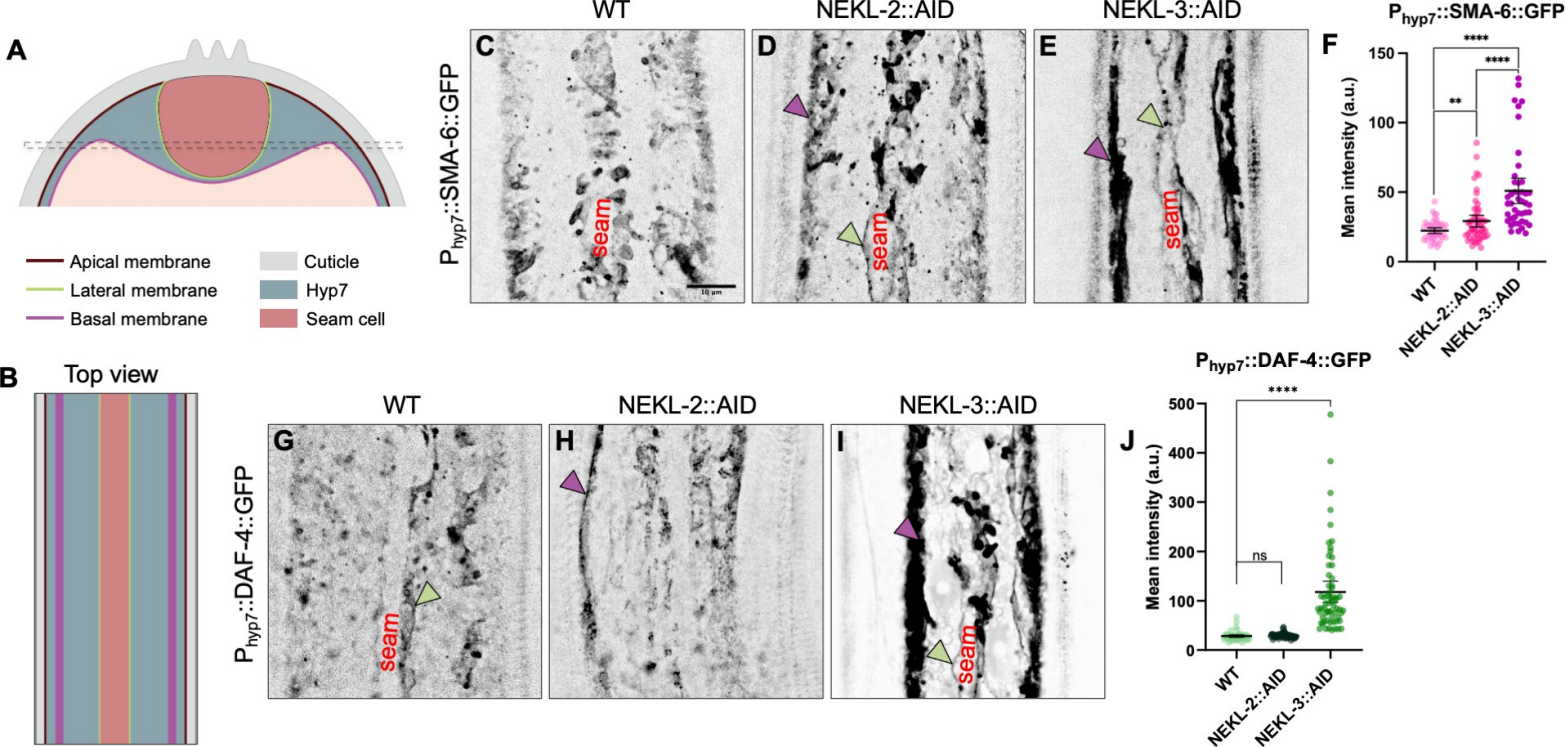
Effects of NEKL-2 or NEKL-3 depletion on basolateral cargoes in *C*. *elegans*. (A) Cross-sectional view of an adult worm depicting the position of the hyp7 syncytium (blue grey) and seam cell syncytium (red). Specific membrane domains of hyp7 are indicated: apical, facing externally directly underneath the cuticle (brown); basal, facing internally (purple); lateral, facing the sides and bottom of the seam cell (green). Dashed lines indicate the medial imaging plane used to acquire the images. (B) Top-down view of the long axis of the worm’s body indicating the positions of the hyp7 and seam cell syncytium along with indicated membranes as in A. (C–E) Representative confocal images of P_hyp-7_::SMA-6::GFP expression in auxin-treated wild-type (C), *nekl-2*::*aid* (D), and *nekl-3*::*aid* (E) day-2 adults. (F, G) Representative confocal images of P_hyp-7_::DAF-4::GFP expression in auxin-treated wild-type (G), *nekl-2*::*aid* (H), and *nekl-3*::*aid* (I) day-2 adults. Green and purple arrowheads (D,E,G) indicate seam and basal membranes (where detectable), respectively. Note that it is often not possible to identify the precise lateral/basolateral boundaries of hyp7 in the SMA-6::GFP and DAF-4::GFP lines in wild type because of the curved nature of the lateral membrane and because of low levels of these cargos marking the membrane. Scale bar in C = 10 μm for C–E, F, and G. (F, J) Mean intensity values for P_hyp-7_::SMA-6::GFP (F) and P_hyp-7_::DAF-4::GFP (J) expression were plotted for individual adults. The two highest datapoints in *nekl-3*::*aid*; DAF-4::GFP (3J; 615 and 1233) were omitted for clarity of presentation. Error bars represent the 95% confidence intervals. Statistical significance was determined using a two-tailed, unpaired t-test: **p < 0.01, ****p < 0.0001. Raw data are available in S1 File.

To investigate possible roles for NEKLs in basolateral endocytosis, we expressed SMA-6::GFP and DAF-4::GFP using a hyp7-specific promoter and examined expression at the membrane of hyp7 adjacent to the seam cell (referred to hereafter as the lateral membrane) and at the membrane that forms the interior boundary of hyp7 (referred to hereafter as the basal membrane; Fig 3A and 3B). Notably, the mean intensity of P_hyp7_::SMA-6::GFP was increased by ~1.3-fold or ~2.3-fold when NEKL-2 or NEKL-3 were depleted, respectively (Fig 3C–3E and 3H). Moreover, a strong increase (~4.8-fold) in the total level of P_hyp7_::DAF-4::GFP was observed after NEKL-3::AID depletion (Fig 3F, 3G and 3I), suggesting that NEKL-3 may also be required for non-clathrin–dependent endocytosis or at the downstream junction of these pathways. In contrast, P_hyp7_::DAF-4::GFP was not detectably affected by depletion of NEKL-2 (Fig 3H and 3J), suggesting that NEKL-2 may not be required for non-CME or may have a more limited role in endocytosis at the basolateral membrane relative to NEKL-3. Accumulation of these markers after NEKL-3 depletion was most evident at or near the basal hyp7 membrane, indicating a defect in plasma membrane uptake and/or processing through the early steps of endocytosis. In addition, some accumulation at or near the basolateral membrane with seam cells was also observed (S4 Fig). Together these results show that NEKL-2 and NEKL-3 play variable roles in the endocytosis of basolateral BMP receptors, although much stronger effects were observed for NEKL-3 in this process.

### NEKLs are required for cargo sorting

Membrane cargoes that are internalized by endocytosis are first delivered to sorting/early endosomes after which they may be recycled back to their original compartment(s) or routed to the lysosome for degradation. Previous studies have characterized the recycling routes for two conserved cargo proteins, MIG-14/Wntless and TGN-38/TGN38, both of which originate from the trans-Golgi [67–71]. MIG-14 is a transmembrane protein that binds to Wnt ligands in the Golgi and is responsible for delivering Wnts to the plasma membrane for secretion. Once at the plasma membrane, MIG-14 is endocytosed and recycled back to the Golgi through a retrograde pathway that requires the retromer complex [72]. In the absence of retromer function, MIG-14 is missorted to the lysosome and is degraded [67,68]. Likewise, TGN-38 is a trans-Golgi resident that is also recycled from the plasma membrane to the trans-Golgi by a retromer-associated retrograde pathway [67,73,74].

**Fig 4:**
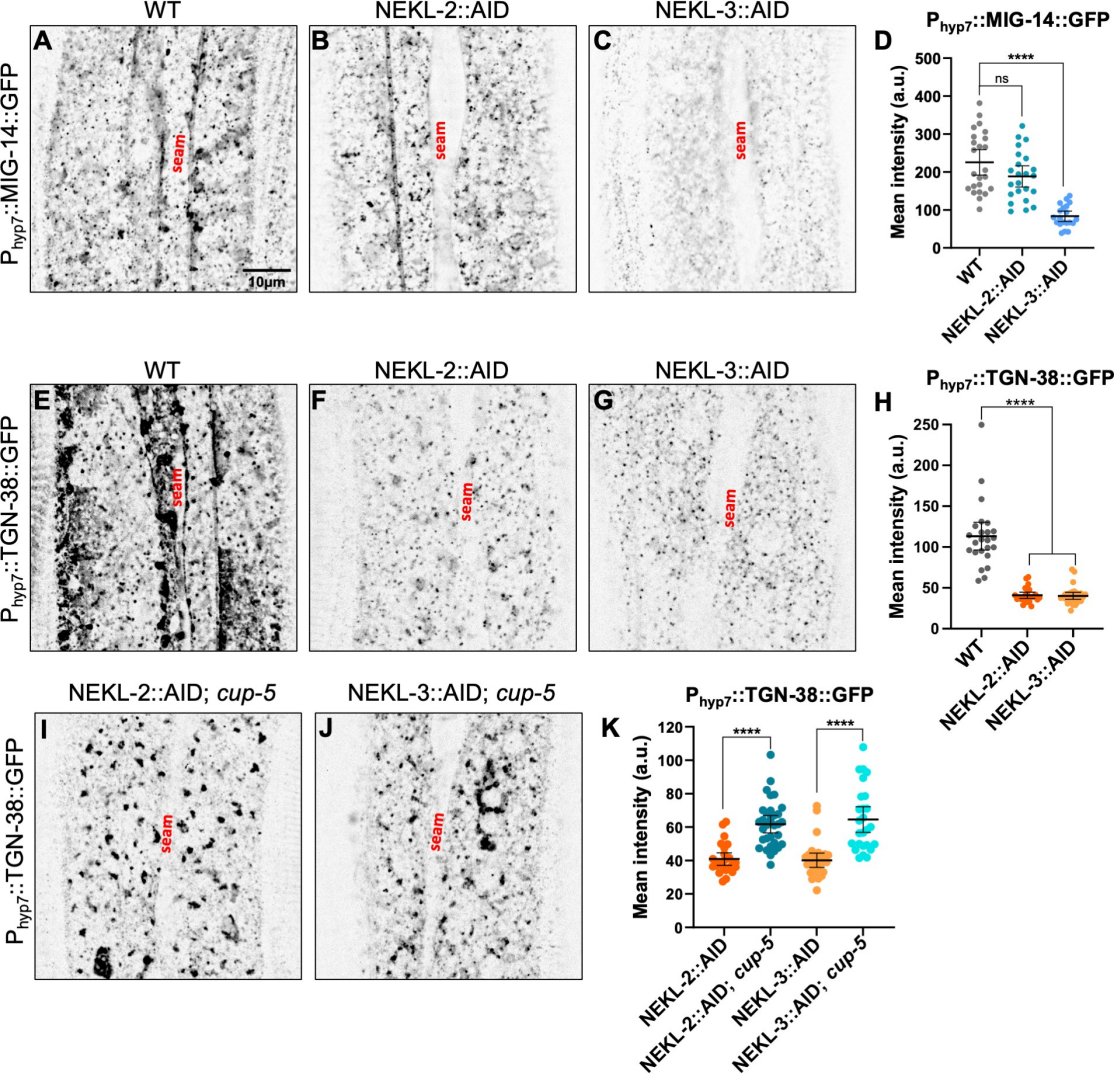
Effects of NEKL-2 or NEKL-3 depletion on cargo sorting in *C*. *elegans*. (A–C, E–G, and I–J) Representative confocal images of P_hyp-7_::MIG-14::GFP (A–C) and P_hyp-7_::TGN-38::GFP (E–G and I–J) expression within hyp7 in auxin-treated wild-type (A, E), NEKL-2::AID (B, F), NEKL-3::AID (C, G), NEKL-2::AID; *cup-5* (I), and NEKL-3::AID; *cup-5* (J) day-2 adults. Scale bar in A = 10 μm for A–C, E–G, I, and J. (D, H) Mean pixel intensity values of P_hyp-7_::MIG-14::GFP and P_hyp-7_::TGN-38::GFP expression for individual worms. Error bars represent the 95% confidence intervals. Statistical significance was determined using a two-tailed, unpaired t-test: ****p < 0.0001; ns, not significant (p > 0.05). Raw data are available in S1 File.

In wild-type adults, both P_hyp7_::MIG-14::GFP and P_hyp7_::TGN-38::GFP markers were observed in punctate structures of varying size throughout the cytoplasm, with some accumulation occurring at the lateral surface (Fig 4A and 4E). After depletion of NEKL-3::AID, P_hyp7_::MIG-14::GFP expression was decreased ~2.7-fold, whereas depletion of NEKL-2::AID resulted in at most a subtle decrease in total P_hyp7_::MIG-14::GFP levels (~1.2-fold; p = 0.09) (Fig 4A–4D). In the case of P_hyp7_::TGN-38::GFP, marker expression was decreased by ~2.8-fold after depletion of either NEKL-2::AID or NEKL-3::AID (Fig 4E–4H). Such findings further highlight differences between NEKL-2 and NEKL-3 with respect to their effects on specific cargoes and suggest that MIG-14::GFP and TGN-38::GFP may be missorted to the lysosomal degradative pathway after loss of NEKLs.

To test if NEKL depletion leads to the aberrant degradation of cargoes, we used CRISPR methods to introduce a partial loss-of-function mutation into *cup-5/trpml1*, which encodes a conserved channel protein required for the maturation of lysosomes and whose loss leads to reduced lysosomal function [75–77]. Notably, reduction of CUP-5 activity led to an ~1.5-fold increase in the total expression of P_hyp7_::TGN-38::GFP in both NEKL-2::AID and NEKL-3::AID depleted strains (Fig 4I–4K). Moreover, accumulation of P_hyp7_::TGN-38::GFP was detected in moderate-to-large internal structures in these strains, which may correspond to defective lysosomes. Together these results indicate that, in addition to functioning in membrane cargo uptake, NEKL-2 and NEKL-3 are also required for the correct sorting and recycling of internalized cargo. We note that loss of *cup-5* in an otherwise wild-type background also led to the accumulation of P_hyp7_::TGN-38::GFP, suggesting that some portion of TGN-38 is normally delivered to the lysosome in wild-type animals and that it’s degradation depends on lysosomal function (S5 Fig). TGN-38 accumulations, however, appeared larger in *cup-5* single mutants relative to NEKL::AID; *cup-5* strains (Figs 4 and S5). One possible explanation for this difference is that NEKL depletion may directly or indirectly affect lysosomal morphology or lysosome abundance.

Depletion of NEKL-3::AID but not NEKL-2::AID also led to a reduction in the levels of P_hyp7_::MIG-14::GFP at the hyp7 membrane surrounding the ALM and PLM neurons, which run longitudinally through hyp7 along the anterior-posterior axis (S6 Fig). Altogether our results indicate that loss of NEKLs leads to a range of defects affecting endosomal compartments as well as cargo internalization and sorting. Moreover, although *nekl-2* and *nekl-3* mutants appear superficially identical with respect to molting defects, they act in largely distinct subcellular compartments and lead to different subcellular phenotypes when depleted.

### The mammalian homologs of NEKL-3, NEK6 and NEK7, are required for normal endocytic trafficking in human cells

**Fig 5:**
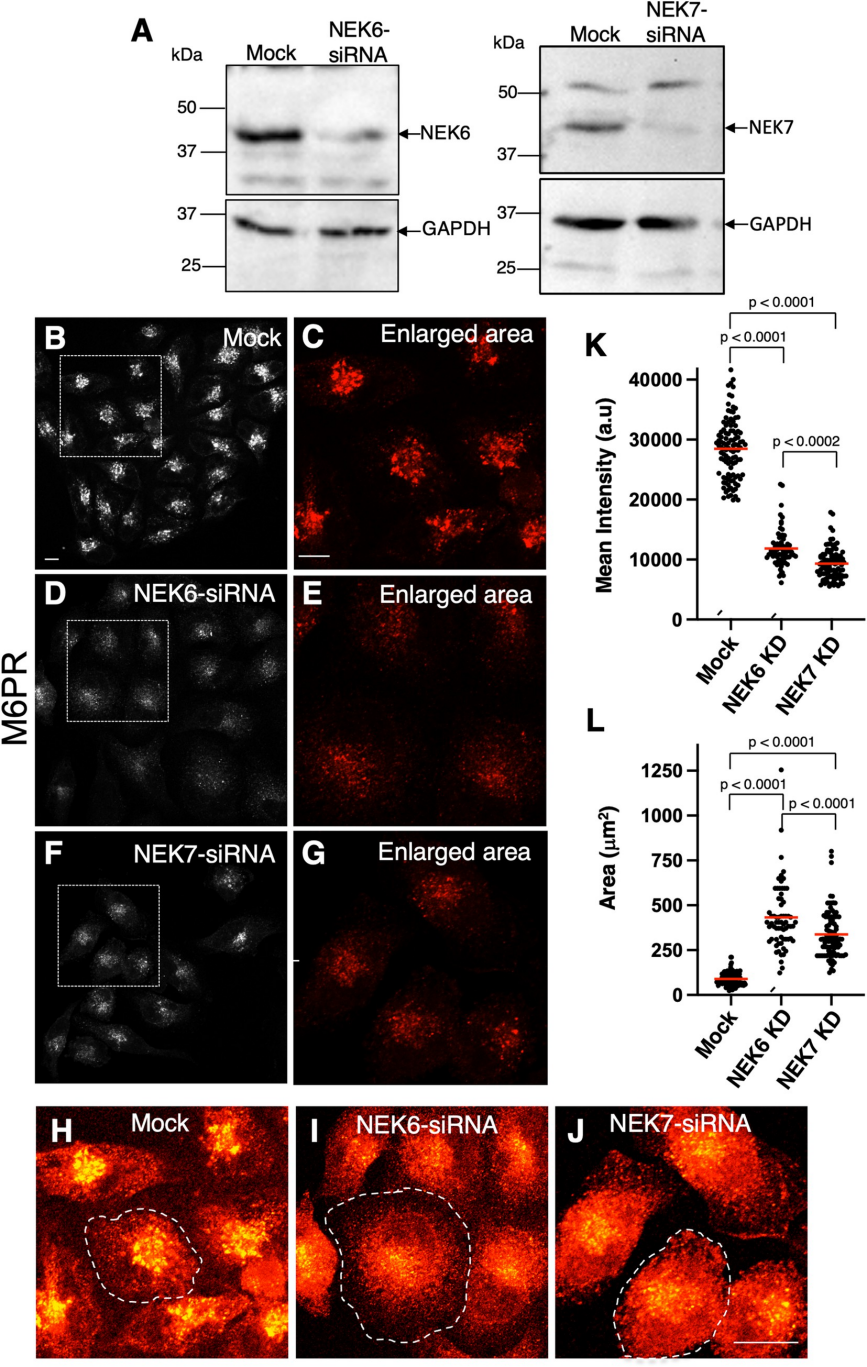
Effects of NEK6 or NEK7 depletion on mannose 6-phosphate receptor trafficking in human cells. (A) siRNA knockdown of NEK6 and NEK7 was validated by western blotting of HeLa cells that were mock-transfected or transfected with oligonucleotides specific for NEK6 (left panel) or NEK7 (right panel). GAPDH was used as the loading control (lower panels). (B–G) Mock-transfected cells (B and enlarged area in C), NEK6 siRNA–transfected cells (D and enlarged area in E), or NEK7 siRNA–transfected cells (F and enlarged area in G) were plated on coverslips and immunostained with antibodies against mannose 6-phosphate receptor (M6PR). (K, L) The mean intensity of M6PR immunostaining (K) and the mean area of M6PR distribution (L) of individual cells in mock-transfected, NEK6 siRNA–transfected, and NEK7 siRNA–transfected cells. (H–J) Saturated and zoomed micrographs demonstrating the distribution and intensity of mannose 6-phosphate receptor immunostaining in mock-transfected (H), NEK6 siRNA–transfected (I), and NEK7 siRNA–transfected cells (J). The dashed lines indicate individual cells. Scale bar in B = 10 μm for B, D, and F; scale bar in C = 10 μm for C, E, and G; scale bar in J = 10 μm for H–J. Statistical significance was determined using Student’s unpaired t-test. Raw data are available in S1 File.

Human NEK6 and NEK7 are both ~70% identical and ~85% similar to NEKL-3 and can rescue molting and trafficking-associated defects when expressed in *C*. *elegans nekl-3* mutants [43]. Nevertheless, a systematic examination of potential roles for NEK6 and NEK7 in intracellular trafficking has not been undertaken. Using siRNA approaches, we were able to substantially reduce protein levels of NEK6 and NEK7 within 48 h of oligonucleotide transfection into cell lines (Fig 5A). As a first measure, we examined the effects of NEK6 and NEK7 knockdown on the cation-independent mannose 6-phosphate receptor (M6PR) in HeLa cells grown at steady state. M6PR is responsible for the delivery of mannose 6-phosphate–tagged lysosomal hydrolases from the trans-Golgi network (TGN), and thus cycles rapidly between the TGN and late endocytic organelles. Accordingly, M6PR is highly sensitive to disruptions in endocytic trafficking, which lead to alterations in its distribution.

Relative to control cells, the mean fluorescence intensity of M6PR-containing endosomes showed a significant decrease (~3-fold) in both NEK6 and NEK7 knockdown cells (Fig 5B–5G and 5K). These findings suggest that, analogous to observations for MIG-14 and TGN-38 in *C*. *elegans*, M6PR may be aberrantly targeted for lysosomal degradation after loss of the NEKL-3 orthologs. In addition, the distribution of M6PR in NEK6/7-depleted cells was altered, with M6PR showing a 2- to 3-fold increase in the area of dispersal (Fig 5H–5J and 5L). Although this increased M6PR dispersal might reflect slightly larger cell sizes in the NEK-depleted cells, M6PR appears to be more homogeneously distributed in the knockdown cells, an effect that has been associated with defects in endosome-to-Golgi transport via the retromer complex [78]. These data imply that both NEK6 and NEK7 are involved in the regulation of endosome-to-Golgi retrograde trafficking.

**Fig 6:**
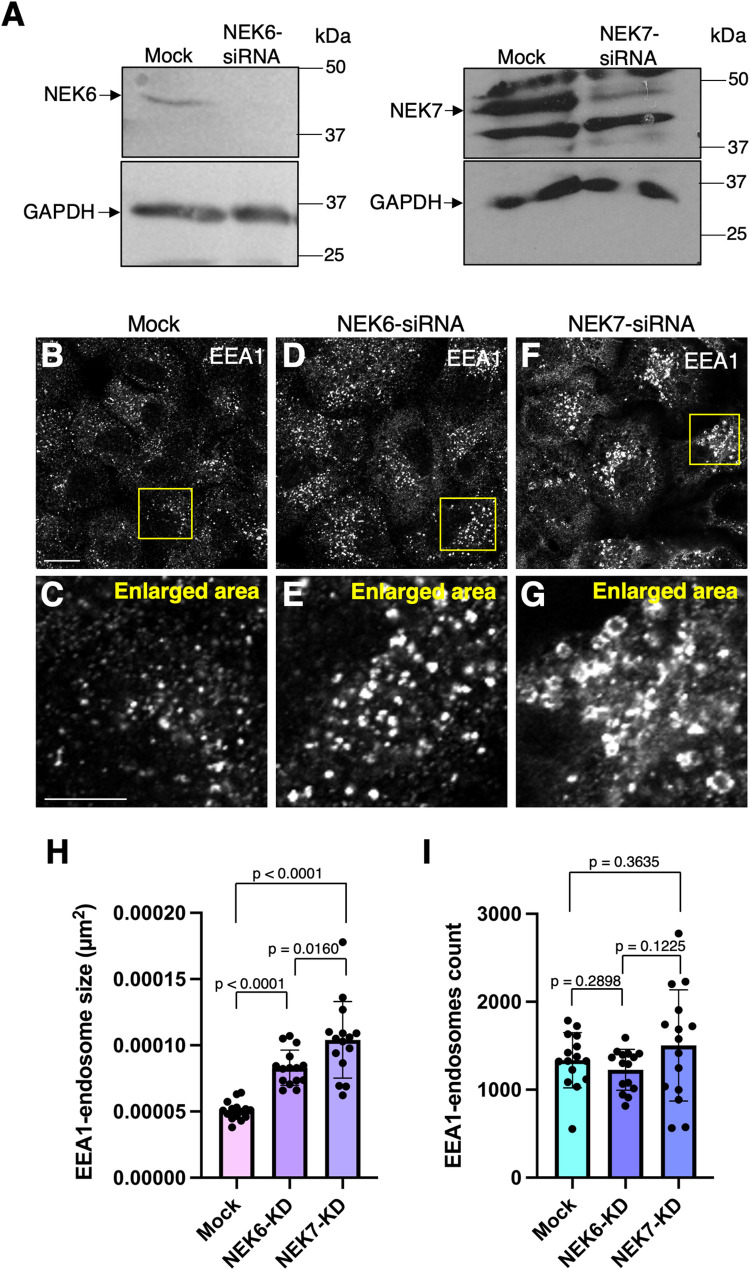
Effects of NEK6 or NEK7 depletion on EEA1-positive sorting endosomes in human cells. (A) siRNA knockdown of NEK6 (left panel) and NEK7 (right panel) in HeLa cells was confirmed by western blotting (B–G) Mock-transfected cells (B and enlarged area in C), NEK6 siRNA–transfected cells (D and enlarged area in E), or NEK7 siRNA–transfected cells (F and enlarged area in G) were plated on coverslips and immunostained with antibodies against the early/sorting endosome marker protein EEA1. Yellow boxes in B, D, and F indicate the area of higher magnification shown in C, E, and G, respectively. (H) The mean size of EEA1-containing endosomes in mock-transfected, NEK6 siRNA–transfected, and NEK7 siRNA–transfected cells. (I) The number of EEA1-containing endosomes in mock-transfected, NEK6 siRNA–transfected, and NEK7 siRNA–transfected cells. Scale bar in B = 10 μm for B, D, and F; Scale bar in C = 5 μm for C, E, and G. Statistical significance was determined using Student’s unpaired t-test. Raw data are available in S1 File.

Given that loss of NEKLs led to a major impact on endosomes, we next assessed the impact of NEK6 and NEK7 depletion on EEA1, a well-characterized early endosomal protein that binds to endosomal PI3P via its FYVE domain [79–81]. Upon siRNA-mediated depletion of NEK6 or NEK7 (Fig 6A) and immunostaining with antibodies against EEA1, endosomal size was increased in cells depleted of NEK6 or NEK7, with those lacking NEK7 displaying especially large ring-like structures decorated by EEA1 (Fig 6B–6G and 6H). In contrast, the number of EEA1 endosomes was not significantly altered upon depletion of either NEK6 or NEK7 (Fig 6I). As a further test, we performed NEK6/7 siRNA knockdown in glioblastoma cells and observed an increase in the size of EEA1-marked vesicles after depletion of NEK7 but not NEK6 (S7 Fig), suggesting cell type–specific requirements for NEK functions. Together, these data suggest a role for NEK6 and NEK7 at endosomes, potentially in the regulation of fusion and/or fission events.

**Fig 7:**
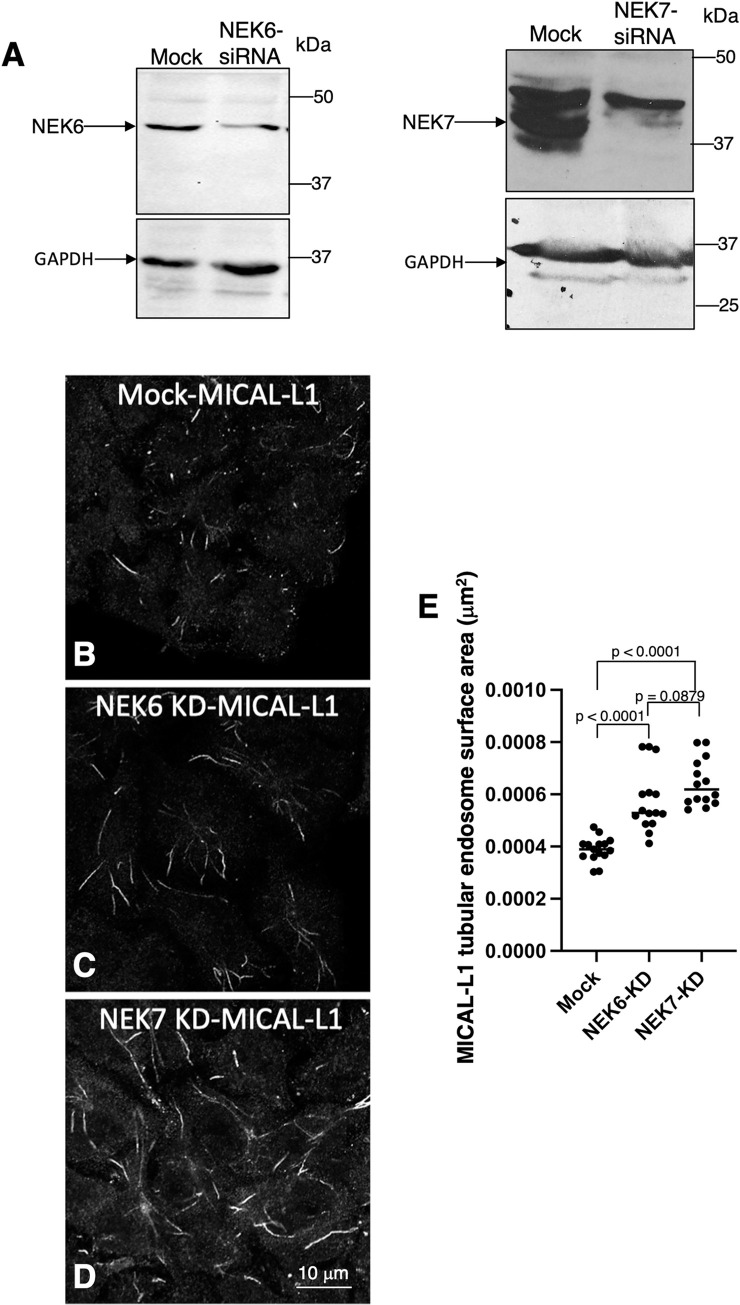
Effects of NEK6 or NEK7 depletion on MICAL-L1–containing tubular recycling endosomes in human cells. (A) siRNA knockdown of NEK6 (left panel) and NEK7 (right panel) was validated by western blotting of HeLa cells. (B–D) Mock-transfected cells (B), NEK6 siRNA–transfected cells (C), or NEK7 siRNA–transfected cells (D) were plated on coverslips and immunostained with antibodies against the tubular recycling endosome marker protein MICAL-L1. (E) The surface area of MICAL-L1–containing endosomes in mock-transfected, NEK6 siRNA–transfected, and NEK7 siRNA–transfected cells. Scale bar in D = 10 μm for B–D. Statistical significance was determined using Student’s unpaired t-test. Raw data are available in S1 File.

To further examine the role of NEK6 and NEK7 at endosomes, we analyzed the effect of their depletion on tubular recycling endosomes marked by the protein MICAL-L1 [82–85]. After depletion of NEK6 or NEK7 with siRNA oligonucleotides (Fig 7A), HeLa cells were fixed and immunostained with antibodies against endogenous MICAL-L1. Inhibition of NEK6 or NEK7 led to an increase in the MICAL-L1 tubular endosomal surface area relative to controls (Fig 7B–7E). Similar observations were observed for glioblastoma cells, with depletion of either NEK6 or NEK7 leading to increased tubulation (S8 Fig). Together, these data indicate roles for NEK6 and NEK7 in endosomal morphology and function in mammalian cells and support the possibility of their involvement in fusion and/or fission activities.

## Discussion

### NIMA kinases are required for proper endosome morphology

In this study we have demonstrated that *C*. *elegans* NEKLs and human NEKs are important for maintaining the proper morphology of endosomal compartments. Loss of NEKL-2 led to enlarged early endosomal compartments with noticeably longer tubular extensions. These changes could indicate a failure of early endosomes to undergo fission, an expansion of early endosomes caused by abnormal cargo retention, or both. Notably, the effect of NEKL-3 loss on early endosomes was distinct from that of NEKL-2, as no increase in tubular extensions was observed. The shape of early endosomes, however, became irregular, and the mean intensity of the early endosomal marker GFP::RAB-5 was increased. This suggests that NEKL-2 and NEKL-3 have distinct roles in regulating early endosome morphology. Notably, siRNA-mediated knockdown of human NEK6 or NEK7 also led to an increase in the size of EEA-1–positive early endosomes in HeLa cells. However, whereas knockdown of NEK7 led to an increase in the size of early endosomes in glioblastoma cells, inhibition of NEK6 did not show this effect, suggesting that NEK6 and NEK7 may regulate early endosomal morphology in a cell type–specific manner.

Consistent with their distinct localization patterns, depletion of NEKL-3 had much stronger effects on late endosomal compartments than did loss of NEKL-2. We note, however, that effects on late endosomes could be due in part to perturbations of early endosomes caused by loss of NEKL-2 and NEKL-3. For technical reasons, we could not determine the expression of NEKLs in recycling endosomes. However, an increase in membrane tubulation was observed with recycling endosomes in hyp7 after loss of NEKL-3 but not NEKL-2. Collectively, these results indicate that NEKL-2 and NEKL-3 have important but distinct roles in regulating endosomal morphology.

### NIMA kinases may broadly affect cargo uptake and recycling

We examined two Golgi residents, MIG-14 and TGN-38, to better understand how NEKLs may influence the endocytic recycling process. We observed a dramatic reduction in the MIG-14 and TGN-38 signal after depletion of NEKL-3. In contrast, loss of NEKL-2 led to a strong reduction in TGN-38, but only minimal effects on MIG-14. These findings suggest that specific cargoes are variably missorted to the lysosomal degradative pathway when NEKL-2 or NEKL-3 is depleted, as was previously reported for mutants affecting actin assembly and the sorting nexin, SNX3 [67,68]. Consistent with this, expression of TGN-38 was rescued when lysosomal function was inhibited in NEKL::AID strains. Interestingly, defects consistent with abnormal recycling were also observed for M6PR when NEK6 and NEK7 were depleted in HeLa cells, consistent with M6PR being incorrectly targeted to lysosomes. Together these findings suggest that NIMA family kinases may have conserved functions in the sorting of cargoes from early endosomes.

Previously, we reported that NEKL-2 and NEKL-3 regulate endocytosis at the apical membrane of hyp7 [43]. In this study, we found that the internalization of a basolateral membrane cargo, SMA-6, was also affected in worms with NEKL-2 or NEKL-3 depletion. Our data also suggest that NEKL-3 may have a role in clathrin-independent endocytosis, as loss of NEKL-3 led to an accumulation of a putative clathrin-independent cargo, DAF-4, near the basolateral surface. One potential caveat to this interpretation is that in the presence of ligand, SMA-6 and DAF-4 would be predicted to form a complex [86], and thus SMA-6 or DAF-4 could potentially affect each other’s uptake or retention. For example, membrane retention of endogenous SMA-6 could impact the localization and uptake of DAF-4::GFP. However, SMA-6 is expressed at very low levels [87,88], and thus it seems unlikely that endogenous plasma-membrane associated SMA-6 could indirectly lead to the strong membrane/juxta-membrane retention of the more highly expressed DAF-4::GFP. Altogether, these findings point to roles for NEKL-3 in both CME and non-CME.

We note that neither SMA-6 nor DAF-4 appeared to be incorrectly targeted to lysosomes after NEKL depletion, which differs from what we observed for MIG-14 and TGN-38. We speculate that SMA-6 and DAF-4 may be trapped at or near the basolateral surface in NEKL-3::AID depleted worms, as our previous study failed to detect clathrin accumulation at basolateral membranes of hyp7 [43]. Moreover, we failed to detect GFP::RAB-5 accumulation at or near basolateral surface in NEKL-3::AID depleted worms, suggesting that the basolateral accumulation of SMA-6 and DAF-4 is not within a RAB-5–marked early endosomal compartment. This buildup SMA-6 and DAF-4 could be due plasma membrane accumulation, internalized juxta-membrane vesicles, or a combination of the two.

### What are the core functions of NEKLs in intracellular trafficking?

In a previous study, we reported that NEKL-2 and NEKL-3 regulate CME at the apical membrane and that they promote the uncoating of clathrin from internalized vesicles [43]. Our current study, however, suggests that the primary sites for trafficking regulation by NEKL-2 and NEKL-3 may be endosomes, and we failed to observe strong colocalization of NEKL-2 or NEKL-3 to AP2–decorated clathrin coated pits and vesicles. Thus, the clathrin-uncoating defects we previously observed could in part be an indirect consequence of endosomal defects. In this context, it is worth noting that RAB-5, which colocalized with both NEKL-2 and NEKL-3, partially localizes to clathrin-coated pits and promotes clathrin and AP2 uncoating [51,89]. Moreover, human RAB5 has been detected at both early endosomes and clathrin-coated vesicles [46,90]. Thus, it remains possible that low levels of NEKL-2 and NEKL-3 may exert direct effects on clathrin uncoating, possibly in conjunction with RAB-5.

The effects we observed on multiple cargoes and compartments indicate that NEKL-2 and NEKL-3 may have widespread functions in intracellular trafficking. Likewise, effects on multiple compartments were observed after human NEK6 and NEK7 knockdowns. This could be due to NIMA kinases regulating distinct targets at different locations within the endocytic network. Alternatively, NIMAs may act on a smaller number of targets, which in turn have widespread functions in trafficking.

In this latter category, one compelling model is that NIMAs could regulate endosomal actin. Actin polymerization is deployed throughout endocytosis to provide mechanical forces needed to bend membranes, facilitate vesicle fission, and promote short-range vesicle transport [91–95]. For example, actin filaments are enriched at sorting endosomes and promote the fission of tubular extensions [52,96]. Actin assembly also plays a role in the sorting of macromolecules from early endosomes and their transport to the trans-Golgi [97,98]. Consistent with this idea, our previous studies showed that actin organization was strongly disrupted in *nekl* mutants and that *nekl* molting defects can be suppressed by loss of function in the conserved actin regulator CDC-42, along with one of its effectors, SID-3/ACK1/2 (activated CDC42 kinase) [38]. Moreover, activated (GTP-bound) CDC-42 was strongly upregulated in *nekl* mutants [38]. Interestingly, based on protein-interaction studies, CDC42 was reported to interact with both NEK6 and NEK7 and could therefore connect the NEKL–MLT network to actin polymerization and to their roles throughout the endocytic network. Future studies will seek to characterize the functional link between NIMA kinases and the actin cytoskeleton, along with their specific roles in regulating endocytosis.

### Human NEKs in endocytosis and cancer

Although human homologs of NEKL-3 (NEK6, NEK7) and NEKL-2 (NEK9) have largely been studied for their roles in cell division, several studies have hinted at potential functions in endocytosis [99–104]. A high-throughput siRNA-based screen of mammalian protein kinases indicated that siRNA-mediated knockdown of NEK6 and NEK7 strongly disrupts CME in HeLa cells [99]. Moreover, genome-wide siRNA studies showed that knockdown of multiple NEK kinases, including NEK6, resulted in the abnormal uptake of endocytic cargoes [101]. A more recent study identified NEK6 as a potential downstream target of NRP-1, which is required for transferrin endocytosis by *Trypanosoma brucei* [105]. Moreover, proteomic studies of NEK6 and NEK7 identified interactions with several trafficking components including alpha and beta subunits of the AP2 adaptor complex (NEK7) [102,103]. Such reports are consistent with our current findings that NEK6 and NEK7 play roles in endocytic trafficking in human cells. Lastly, our ability to rescue both molting and trafficking defects through the expression of human NEKs in *C*. *elegans nekl* mutants suggests that these functions are conserved [43].

Finally, our studies provide evidence that NEKLs may affect two highly conserved signaling pathways, BMP and Wnt, which are misregulated in many human cancers [106–108]. Previous studies have described the role of endocytosis in cancers including how endocytosis adapts to the needs of cancer cells, a phenomenon known as “adaptive CME” [109–111]. For example, upregulation of clathrin light-chain (CLCb) leads to an increased rate of CME; this in turn alters the trafficking of oncogenic epidermal growth factor receptor (EGFR) and promotes cancer cell migration and metastasis [112]. Thus, it is possible that NIMA kinases, which are overexpressed in many tumors, could be driving cancer formation in part through their roles in trafficking as well as by their previously described functions in mitosis [19,113–117]. Future studies will elucidate how NIMA kinases control trafficking, which will aid in understanding their involvement in human disease.

## Materials and methods

### Strains and maintenance

*C*. *elegans* strains were maintained according to standard protocols [118] and were propagated at 22°C unless stated otherwise. Strains used in this study are listed in S1 Table. Loss-of-function alleles of *cup-5* (*fd395* and *fd397*) were generated by the CRISPR-Cas9 protocol described by Ghanta et al. [119]. Sequences for the sgRNA and repair template are given below. Capital letters represent the altered or inserted nucleotides.

sgRNA: 5′-aacgatgcgcttattatcat-3′

Repair Template: 5′-aacttaaatttttataaaaattacctgaaactcgatggatatttttgcaacGGTCTCaatgataataagcgcatcgttgaccacaatcatcacataccacaaattcag-3′

### Reporter strain construction

Plasmids for *C*. *elegans* hyp7-specific expression used the promoter from *semo-1/Y37A1B*.*5* (P_hyp-7_) [120]. Vector details are available upon request. Cloning was performed using the Gateway system (Invitrogen) and modified versions of hygromycin-resistant and miniMos-enabled vector pCFJ1662 (gift from Erik Jorgensen, University of Utah; Addgene #51482). pDONR221 entry vectors containing coding regions for *rab-5*, *rab-7*, *mig-14*, *tgn-38*, *aman-2*, *daf-4*, and *sma-6* were transferred into hyp7 destination vectors with the Gateway LR clonase II reaction to generate C-/N-terminal fusions. Single-copy integrations were obtained using miniMos technology [121].

### Image acquisition

Confocal fluorescence images in Figs 1–4 and S1 and S3 and S5 were acquired using an Olympus IX83 inverted microscope with a Yokogawa spinning-disc confocal head (CSU-W1). z-Stack images were acquired using a 100×, 1.35 N.A. silicone oil objective. cellSense 3.1 software (Olympus Corporation) was used for image acquisition. Fluorescence images in S2 Fig were acquired using an Olympus IX81 inverted microscope with a Yokogawa spinning-disc confocal head (CSU-X1). MetaMorph 7.7 software was used for image acquisition. z-Stack images were acquired using a 100×, 1.40 N.A. oil objective.

Confocal images for Figs 5–7 and S6 and S7 were acquired using an Olympus IX83 inverted microscope with a Yokogawa spinning-disc confocal head (CSU-W1) or with a Zeiss LSM 800 microscope with a 63×, 1.40 N.A. objective with appropriate filters.

### Image analysis

Mean intensity (measured in arbitrary units, a.u.), quantification of various shape parameters, and the colocalization analysis were performed using Fiji software [122]. To quantify mean intensity (Figs 2D, 2J, 2P, 3H, 3I, 4D, 4H, 4K and S3B) for a z-plane of interest, rolling ball background subtraction was performed (radius = 50 pixels), and the polygon selection tool was used to choose the region of hyp7 in which the mean intensity was quantified.

To quantify shape parameters, a z-plane of interest was selected, and the minimum filter (radius = 10.0 pixels) was applied to the raw image. The filter-applied image was then subtracted from the raw image using the image calculator function. Images were then thresholded, and the Despeckle function was used to remove noise corresponding to 1 pixel in size. Finally, various shape parameters were quantified using the Shape Descriptor plugin.

For colocalization studies, the raw z-stack images were deconvoluted using the Wiener deconvolution algorithm (cellSense 3.1 software). The desired z-plane was extracted from both deconvoluted and raw z-stack images. Then, the Gaussian filter (radius = 10 pixels) was applied to the deconvoluted image, which was subtracted from the original deconvoluted image using the image calculator function. Next, these subtracted images were thresholded to obtain binary images to be used as masks. These binary masks were combined using the “AND” Boolean operation to the background-subtracted (rolling ball algorithm; radius = 50 pixels) raw images. Using the polygon tool, the region of hyp7 was selected in the combined images, and coloc2 plugin was used to calculate Manders’ coefficient.

The mean area and intensity of fluorescence in HeLa cells Fig 5K and 5L was obtained with Zeiss LSM Zen software, after outlining a region of interest. EEA1-positive endosome size (Fig 6H) was measured using Imaris software, and MICAL-L1 tubular endosome surface area (Fig 7E) was quantified using ImageJ software., or the number of MICAL-L1 tubular recycling endosomes were counted manually (S8 Fig), since number and area are generally correlated. Endosome size in the human cell studies was quantified using NIH ImageJ. Size parameters were set for 0 –infinity. Brightness parameters were selected to eliminate recognition of background by ImageJ’s particle counter while optimizing selection of true positive fluorescent pixels. Statistical analyses was performed in Prism by first testing the assumption of normal distribution with the D’Agostino and Pearson normality test. Statistical significance was then calculated with an unpaired two-tailed t-test.

### Auxin treatment

Auxin (indole-3-acetic acid) was purchased from Alfa Aesar. A 100× stock auxin solution (0.4 M) was made by dissolving 0.7 g of auxin in 10 ml of 100% ethanol. A mixture of 25 μl of stock auxin solution and 225 μl of autoclaved deionized water was added to each plate containing day-1 adult worms.

### Antibodies

Rabbit antibodies against NEK6 were obtained from Mybiosource (Cat# MBS94186), rabbit antibodies against NEK7 were obtained from Novus (Cat# 31110), mouse horseradish peroxidase (HRP)-conjugated antibodies against GAPDH were obtained from Proteintech (Cat# HRP-60004), rabbit antibodies against EEA1 were obtained from Cell Signaling (Cat# 3288), and mouse polyclonal antibodies against MICAL-L1 were obtained from Novus (Cat# H00085777-B01P).

### Immunoblotting

HeLa cells were lysed in lysis buffer containing 50 mM Tris-HCl (pH 7.4), 150 mM NaCl, 1% NP-40, and 0.5% deoxycholate. Proteins from the lysates were separated by SDS-PAGE, transferred to nitrocellulose filters, and immunoblotted with antibodies using standard methods.

### siRNA knockdown

Cells were transfected with siRNA oligonucleotides from Sigma (600 nM NEK6 600 or 100 nM NEK7) using Dharmafect transfection reagent (Dharmacon) for 48 h, prior to validation of knockdown by immunoblotting using GAPDH as the loading control as described above.

### Statistics

All statistical tests were performed using software from Prism GraphPad.

## Supporting information

S1 Fig Colocalization between NEKL-2 and NEKL-3. Colocalization assays were carried out in adult worms expressing both NEKL-2::mNeonGreen and NEKL-3::mKate. (A–C, A’–C’, D–F, and D’–F’) Representative images of the apical region of hyp7 in adult worms expressing NEKL-2::mNeonGreen and NEKL-3::mKate (A–C and A’–C’) as well as the medial plane of hyp7 (D–F and D’–F’). Here the medial plane is considered the plane ~1 μm below the apical surface. Scale bar in A = 10 μm for A–F. Scale bar in A’ = 1 μm for A’–F’. (G, H) Manders’ coefficient was calculated and plotted for individual worms in *nekl-2*::*mNeonGreen*; *nekl-3*::*mKate* strains. The fraction of NEKL-2::mNeonGreen puncta overlapping with NEKL-3::mKate puncta in the apical and medial plane (G) and vice versa (H) are shown. Raw data are available in S1 File.
(TIFF)

S2 Fig Colocalization of NEKLs with clathrin-coated pits. (A–F) Colocalization assays were performed on strains expressing either NEKL-2::mNeonGreen (A–C) or NEKL-3::mNeonGreen (D–F) with APA-2::mScarlet, the alpha subunit of the AP2 adaptor complex, which is present in clathrin-coated pits. Note that these images were collected on a different confocal microscope and thus appear somewhat different than images in the paper (see Materials and Methods). Scale bar in A = 10 μm in A–F.
(TIFF)

S3 Fig Effects of NEKL depletion on Golgi compartments. (A) Representative confocal images of P_hyp7_::AMAN-2::mNeonGreen expression in the indicated backgrounds. (B, C) Mean intensity of P_hyp7_::AMAN-2::mNeonGreen expression (B) and the mean number of P_hyp7_::AMAN-2::mNeonGreen-positive vesicles (C) for individual worms were plotted in the graphs. (D) Representative confocal images of P_hyp7_::AMAN-2::mNeonGreen co-expressed with a functional multi-copy NEKL-3::mCherry reporter. Error bars represent the 95% confidence intervals. p-Values were obtained by comparing means using an unpaired t-test: ****p < 0.0001, ***p < 0.001; ns, not significant (p > 0.05). Raw data are available in S1 File.
(TIFF)

S4 Fig Three-dimensional rendering of effects of NEKL deletion on basolateral cargoes. (A–C) Three-dimensional plots showing the individual pixel intensity along the *x* and *y* planes for the fluorescent images presented in Fig 3C–3E. (D,E) Three-dimensional plots showing the pixel intensity value along the *x* and *y* planes for the fluorescent images presented in Fig 3F and 3G. We note that the basal boundary occurs in the *x* axes at approximately 40 μm and 10 μm whereas the seam cell boundary occurs between 20–30 μm.
(TIFF)

S5 Fig Effects of cup-5 loss of function on a membrane cargo, TGN-38. Representative images of P_hyp-7_::TGN-38::GFP expression in auxin-treated day-2 adults in wildtype and *cup-5* mutant background. Red arrows indicate accumulation of cargoes in vesicle-like structures. Mean intensity values of the P_hyp-7_::TGN-38::GFP expression for individual worms are plotted in the graph. Error bars represent the 95% confidence intervals. p-Values were obtained by comparing means using an unpaired t-test: ****p < 0.0001, ns, not significant (p > 0.05). Raw data are available in S1 File.
(TIFF)

S6 Fig Effects of NEKL depletion on cargo trafficking near the neural membrane. Representative images of P_hyp-7_::MIG-14::GFP expression in auxin-treated wild-type, *nekl-2*::*aid*, and *nekl-3*::*aid* day-2 adults in the apical/medial plane. Red arrows show the presence of MIG-14::GFP expression in wild-type and *nekl-2*::*aid* adults at the hyp7 membrane surrounding the ALM neuron. The red arrowhead indicates the absence of MIG-14::GFP expression in *nekl-3*::*aid* worms. Scale bar = 10 μm in the three lower-magnification images. The yellow bracket along the ALM neuron in wild type indicates the region show in the higher magnification image (yellow outline). The bar graph shows the percentage of worms exhibiting P_hyp-7_::MIG-14::GFP expression surrounding the ALM/PLM neurons in the indicated backgrounds. Numbers of worms are indicated for each bar. Error bars represent the 95% confidence interval. p-Values were obtained using Fisher’s exact test: ****p < 0.0001. Raw data are available in S1 File.
(TIFF)

S7 Fig Effect of NEK6 or NEK7 depletion in early endosomal compartments in glioblastoma cells. Mock-transfected cells, NEK6 siRNA-transfected cells, or NEK7 siRNA-transfected cells were plated on coverslips and immunostained with antibodies against the sorting endosome marker protein EEA1. Average sizes of early/sorting endosomes are shown for the three conditions, with significant differences observed between the mock-transfected cells and both siRNA treatments as well as between NEK6 siRNA and NEK7 siRNA-transfected cells. Yellow box in panels indicates region of enlarged inset. Scale bars = 10 μm.
(TIFF)

S8 Fig Effects of NEK6 or NEK7 depletion on recycling endosomes in glioblastoma cells. Glioblastoma cells were mock transfected or were transfected with NEK6 siRNA or NEK7 siRNA. Transfected cells were then plated on coverslips and immunostained with antibodies against the tubular recycling endosome marker protein MICAL-L1. Average numbers of MICAL-L1 tubular endosomes per field were determined for the three conditions, with significant differences observed between the mock-transfected cells and both siRNA treatments. Yellow box in panels indicates region of enlarged inset. Scale bars = 10 μm.
(TIFF)

S1 Table List of all the strains used in this study. (PDF)

S1 File Compilation of raw data used in this study. (XLSX)

## References

[1] Oakley B, Morris R. A mutation in aspergillus nidulans that blocks the transition from interphase to prophase. Journal of Cell Biology. 1983 Apr 1;96(4):1155–8. doi: 10.1083/jcb.96.4.1155 6339527 PMC2112314

[2] Osmani SA, Pu RT, Morris NR. Mitotic induction and maintenance by overexpression of a G2-specific gene that encodes a potential protein kinase. Cell. 1988;53(2):237–44. doi: 10.1016/0092-8674(88)90385-6 3359487

[3] Osmani AH, McGuire SL, Osmani SA. Parallel activation of the NIMA and p34cdc2 cell cycle-regulated protein kinases is required to initiate mitosis in A. nidulans. Cell. 1991;67(2):283–91. doi: 10.1016/0092-8674(91)90180-7 1913824

[4] Pu RT, Osmani SA. Mitotic destruction of the cell cycle regulated NIMA protein kinase of Aspergillus nidulans is required for mitotic exit. EMBO Journal. 1995;14(5):995–1003. doi: 10.1002/j.1460-2075.1995.tb07080.x 7889945 PMC398171

[5] Roig J, Groen A, Caldwell J, Avruch J. Active Nercc1 Protein Kinase Concentrates at Centrosomes Early in Mitosis and Is Necessary for Proper Spindle Assembly. Mol Biol Cell. 2005 Oct;16(10):4827–40. doi: 10.1091/mbc.e05-04-0315 16079175 PMC1237086

[6] Fry AM O’Regan L, Sabir SR, Bayliss R. Cell cycle regulation by the NEK family of protein kinases. J Cell Sci. 2012;125(19):4423–33. doi: 10.1242/jcs.111195 23132929 PMC3500863

[7] Fry AM, Bayliss R, Roig J. Mitotic regulation by NEK kinase networks. Front Cell Dev Biol. 2017;5(DEC):1–13. doi: 10.3389/fcell.2017.00102 29250521 PMC5716973

[8] Moniz L, Dutt P, Haider N, Stambolic V. Nek family of kinases in cell cycle, checkpoint control and cancer. Cell Div. 2011 Dec 31;6(1):18. doi: 10.1186/1747-1028-6-18 22040655 PMC3222597

[9] Brieño-Enríquez MA, Moak SL, Holloway JK, Cohen PE. NIMA-related kinase 1 (NEK1) regulates meiosis I spindle assembly by altering the balance between α-Adducin and Myosin X. PLoS One. 2017;12(10):1–18. 10.1371/journal.pone.0185780 PMC5628868 28982183

[10] Fry AM. The Nek2 protein kinase: A novel regulator of centrosome structure. Oncogene. 2002;21(40 REV. ISS. 4):6184–94. doi: 10.1038/sj.onc.1205711 12214248

[11] Prosser SL, Sahota NK, Pelletier L, Morrison CG, Fry AM. Nek5 promotes centrosome integrity in interphase and loss of centrosome cohesion in mitosis. Journal of Cell Biology. 2015;209(3):339–48. doi: 10.1083/jcb.201412099 25963817 PMC4427792

[12] Yissachar N, Salem H, Tennenbaum T, Motro B. Nek7 kinase is enriched at the centrosome, and is required for proper spindle assembly and mitotic progression. FEBS Lett. 2006;580(27):6489–95. doi: 10.1016/j.febslet.2006.10.069 17101132

[13] Bertran MT, Sdelci S, Regué L, Avruch J, Caelles C, Roig J. Nek9 is a Plk1-activated kinase that controls early centrosome separation through Nek6/7 and Eg5. EMBO Journal. 2011;30(13):2634–47. doi: 10.1038/emboj.2011.179 21642957 PMC3155310

[14] O’Connell MJ, Krien MJE, Hunter T. Never say never. The NIMA-related protein kinases in mitotic control. Trends Cell Biol. 2003;13(5):221–8. doi: 10.1016/s0962-8924(03)00056-4 12742165

[15] Prosser SL O’Regan L, Fry AM. Novel insights into the mechanisms of mitotic spindle assembly by NEK kinases. Mol Cell Oncol. 2016;3(3):1–4. doi: 10.1080/23723556.2015.1062952 27314078 PMC4909405

[16] Bachus S, Graves D, Fulham L, Akkerman N, Stephanson C, Shieh J, et al. In Mitosis You Are Not: The NIMA Family of Kinases in Aspergillus, Yeast, and Mammals. Int J Mol Sci. 2022;23(7). doi: 10.3390/ijms23074041 35409400 PMC8999480

[17] Bian Z, Liao H, Zhang Y, Wu Q, Zhou H, Yang Z, et al. Never in mitosis gene a related kinase-6 attenuates pressure overload-induced activation of the protein kinase B pathway and cardiac hypertrophy. PLoS One. 2014;9(4):1–8. doi: 10.1371/journal.pone.0096095 24763737 PMC3999101

[18] Bowers AJ, Boylan JF. Nek8, a NIMA family kinase member, is overexpressed in primary human breast tumors. Gene. 2004;328(1–2):135–42. doi: 10.1016/j.gene.2003.12.002 15019993

[19] He Z, Ni X, Xia L, Shao Z. Overexpression of NIMA-related kinase 6 (NEK6) contributes to malignant growth and dismal prognosis in Human Breast Cancer. Pathol Res Pract. 2018;214(10):1648–54. doi: 10.1016/j.prp.2018.07.030 30153958

[20] Hayward DG, Fry AM. Nek2 kinase in chromosome instability and cancer. Cancer Lett. 2006;237(2):155–66. doi: 10.1016/j.canlet.2005.06.017 16084011

[21] He Y, Zeng MY, Yang D, Motro B, Núñez G. NEK7 is an essential mediator of NLRP3 activation downstream of potassium efflux. Nature. 2016;530(7590):354–7. doi: 10.1038/nature16959 26814970 PMC4810788

[22] Jeltema D, Wang J, Cai J, Kelley N, Yang Z, He Y. A Single Amino Acid Residue Defines the Difference in NLRP3 Inflammasome Activation between NEK7 and NEK6. The Journal of Immunology. 2022;208(8):2029–36. doi: 10.4049/jimmunol.2101154 35354613 PMC9012696

[23] Cohen S, Aizer A, Shav-Tal Y, Yanai A, Motro B. Nek7 kinase accelerates microtubule dynamic instability. Biochim Biophys Acta Mol Cell Res. 2013;1833(5):1104–13. doi: 10.1016/j.bbamcr.2012.12.021 23313050

[24] Otto EA, Trapp ML, Schultheiss UT, Helou J, Quarmby LM, Hildebrandt F. NEK8 mutations affect ciliary and centrosomal localization and may cause nephronophthisis. Journal of the American Society of Nephrology. 2008;19(3):587–92. doi: 10.1681/ASN.2007040490 18199800 PMC2391043

[25] Casey JP, Brennan K, Scheidel N, McGettigan P, Lavin PT, Carter S, et al. Recessive NEK9 mutation causes a lethal skeletal dysplasia with evidence of cell cycle and ciliary defects. Hum Mol Genet. 2016;25(9):1824–35. doi: 10.1093/hmg/ddw054 26908619

[26] Choi HJC, Lin JR, Vannier JB, Slaats GG, Kile AC, Paulsen RD, et al. NEK8 links the ATR-regulated replication stress response and S phase CDK activity to renal ciliopathies. Mol Cell. 2013;51(4):423–39. doi: 10.1016/j.molcel.2013.08.006 23973373 PMC3790667

[27] Thiel C, Kessler K, Giessl A, Dimmler A, Shalev SA, Von Der Haar S, et al. NEK1 mutations cause short-rib polydactyly syndrome type majewski. Am J Hum Genet. 2011;88(1):106–14. doi: 10.1016/j.ajhg.2010.12.004 21211617 PMC3014367

[28] Zalli D, Bayliss R, Fry AM. The Nek8 protein kinase, mutated in the human cystic kidney disease nephronophthisis, is both activated and degraded during ciliogenesis. Hum Mol Genet. 2012;21(5):1155–71. doi: 10.1093/hmg/ddr544 22106379 PMC3277313

[29] Grampa V, Delous M, Zaidan M, Odye G, Thomas S, Elkhartoufi N, et al. Novel NEK8 Mutations Cause Severe Syndromic Renal Cystic Dysplasia through YAP Dysregulation. PLoS Genet. 2016;12(3):1–32. doi: 10.1371/journal.pgen.1005894 26967905 PMC4788435

[30] Coene KLM, Mans DA, Boldt K, Gloeckner CJ, van Reeuwijk J, Bolat E, et al. The ciliopathy-associated protein homologs RPGRIP1 and RPGRIP1L are linked to cilium integrity through interaction with Nek4 serine/threonine kinase. Hum Mol Genet. 2011;20(18):3592–605. doi: 10.1093/hmg/ddr280 21685204

[31] Ding XF, Chen J, Zhou J, Chen G, Wu YL. Never-in-mitosis a-related kinase 8, a novel targof von-hippel-lindau tumor suppressor protein, promotes gastric cancer cell proliferation. Oncol Lett. 2018;16(5):5900–6. 30333866 10.3892/ol.2018.9328 PMC6176424

[32] Abeyta A, Castella M, Jacquemont C, Taniguchi T. NEK8 regulates DNA damage-induced RAD51 foci formation and replication fork protection. Cell Cycle. 2017;16(4):335–47. doi: 10.1080/15384101.2016.1259038 27892797 PMC5324754

[33] Yamamoto Y, Chino H, Tsukamoto S, Ode KL, Ueda HR, Mizushima N. NEK9 regulates primary cilia formation by acting as a selective autophagy adaptor for MYH9/myosin IIA. Nat Commun. 2021;12(1):1–16. 34078910 10.1038/s41467-021-23599-7 PMC8172835

[34] Yochem J, Lažetić V, Bell L, Chen L, Fay D. C. elegans NIMA-related kinases NEKL-2 and NEKL-3 are required for the completion of molting. Dev Biol. 2015;398(2):255–66. 25523392 10.1016/j.ydbio.2014.12.008 PMC4314388

[35] Hoff S, Halbritter J, Epting D, Frank V, Nguyen TMT, van Reeuwijk J, et al. ANKS6 is a central component of a nephronophthisis module linking NEK8 to INVS and NPHP3. Nat Genet. 2013 Aug 23;45(8):951–6. doi: 10.1038/ng.2681 23793029 PMC3786259

[36] Czarnecki PG, Gabriel GC, Manning DK, Sergeev M, Lemke K, Klena NT, et al. ANKS6 is the critical activator of NEK8 kinase in embryonic situs determination and organ patterning. Nat Commun. 2015;6(May 2014). doi: 10.1038/ncomms7023 25599650 PMC4361001

[37] Ramachandran H, Engel C, Müller B, Dengjel J, Walz G, Yakulov TA. Anks3 alters the sub-cellular localization of the Nek7 kinase. Biochem Biophys Res Commun. 2015;464(3):901–7. doi: 10.1016/j.bbrc.2015.07.063 26188091

[38] Lažetić V, Joseph BB, Bernazzani SM, Fay DS. Actin organization and endocytic trafficking are controlled by a network linking NIMA-related kinases to the CDC-42-SID-3/ACK1 pathway. PLoS Genet. 2018;14(4):1–30. 10.1371/journal.pgen.1007313 PMC5897031 29608564

[39] Lažetić V, Fay DS. Conserved ankyrin repeat proteins and their NIMA kinase partners regulate extracellular matrix remodeling and intracellular trafficking in Caenorhabditis elegans. Genetics. 2017;205(1):273–93. doi: 10.1534/genetics.116.194464 27799278 PMC5223508

[40] Binti S, Melinda R V., Joseph BB, Edeen PT, Miller SD, Fay DS. A life cycle alteration can correct molting defects in Caenorhabditis elegans. Dev Biol. 2022;483(December 2021):143–56. doi: 10.1016/j.ydbio.2022.01.001 35038442 PMC8867747

[41] Lažetić V, Fay DS. Molting in C. elegans. Worm. 2017 Jan 2;6(1):e1330246. doi: 10.1080/21624054.2017.1330246 28702275 PMC5501215

[42] Larval C, Miao R, Li M, Zhang Q, Miao R, Li M, et al. An ECM-to-Nucleus Signaling Pathway Activates Article An ECM-to-Nucleus Signaling Pathway Activates Lysosomes for C. elegans Larval Development. Dev Cell. 2020;52(1):21–37.e5. 31735670 10.1016/j.devcel.2019.10.020

[43] Joseph BB, Wang Y, Edeen P, Lazetic V, Grant BD, Fay DS. Control of clathrin-mediated endocytosis by NIMA family kinases. Vol. 16, PLoS Genetics. 2020. 1–38 p. doi: 10.1371/journal.pgen.1008633 32069276 PMC7048319

[44] Yochem J, Tuck S, Greenwald I, Han M. A gp330/megalin-related protein is required in the major epidermis of Caenorhabditis elegans for completion of molting. Development. 1999 Feb 1;126(3):597–606. doi: 10.1242/dev.126.3.597 9876188

[45] Herz J, Strickland DK. LRP: a multifunctional scavenger and signaling receptor. Journal of Clinical Investigation. 2001 Sep 15;108(6):779–84. doi: 10.1172/JCI13992 11560943 PMC200939

[46] Bucci C, Parton RG, Mather IH, Stunnenberg H, Simons K, Hoflack B, et al. The small GTPase rab5 functions as a regulatory factor in the early endocytic pathway. Cell. 1992;70(5):715–28. doi: 10.1016/0092-8674(92)90306-w 1516130

[47] Poteryaev D, Datta S, Ackema K, Zerial M, Spang A. Identification of the switch in early-to-late endosome transition. Cell. 2010;141(3):497–508. doi: 10.1016/j.cell.2010.03.011 20434987

[48] Hyttinen JMT, Niittykoski M, Salminen A, Kaarniranta K. Maturation of autophagosomes and endosomes: A key role for Rab7. Biochim Biophys Acta Mol Cell Res. 2013;1833(3):503–10. doi: 10.1016/j.bbamcr.2012.11.018 23220125

[49] Bucci C, Thomsen P, Nicoziani P, McCarthy J, van Deurs B. Rab7: A Key to Lysosome Biogenesis. Pfeffer SR, editor. Mol Biol Cell. 2000 Feb;11(2):467–80. doi: 10.1091/mbc.11.2.467 10679007 PMC14786

[50] Woodman PG. Biogenesis of the sorting endosome: The role of Rab5. Traffic. 2000;1(9):695–701. doi: 10.1034/j.1600-0854.2000.010902.x 11208157

[51] Semerdjieva S, Shortt B, Maxwell E, Singh S, Fonarev P, Hansen J, et al. Coordinated regulation of AP2 uncoating from clathrin-coated vesicles by rab5 and hRME-6. Journal of Cell Biology. 2008;183(3):499–511. doi: 10.1083/jcb.200806016 18981233 PMC2575790

[52] Gautreau A, Oguievetskaia K, Ungermann C. Function and regulation of the endosomal fusion and fission machineries. Vol. 6, Cold Spring Harbor Perspectives in Biology. 2014. doi: 10.1101/cshperspect.a016832 24591520 PMC3949357

[53] Klumperman J, Raposo G. The Complex Ultrastructure of the Endolysosomal System. Cold Spring Harb Perspect Biol. 2014 Oct 1;6(10):a016857–a016857. doi: 10.1101/cshperspect.a016857 24851870 PMC4176003

[54] Jovic M, Sharma M, Rahajeng J, Caplan S. The early endosome: A busy sorting station for proteins at the crossroads. Histol Histopathol. 2010;25(1):99–112. doi: 10.14670/HH-25.99 19924646 PMC2810677

[55] Mayor S, Presley JF, Maxfield FR. Sorting of membrane components from endosomes and subsequent recycling to the cell surface occurs by a bulk flow process. Journal of Cell Biology. 1993;121(6):1257–69. doi: 10.1083/jcb.121.6.1257 8509447 PMC2119709

[56] Holland AJ, Fachinetti D, Han JS, Cleveland DW. Inducible, reversible system for the rapid and complete degradation of proteins in mammalian cells. Proc Natl Acad Sci U S A. 2012;109(49). doi: 10.1073/pnas.1216880109 23150568 PMC3523849

[57] Zhang L, Ward JD, Cheng Z, Dernburg AF. The auxin-inducible degradation (AID) system enables versatile conditional protein depletion in C. elegans. Development (Cambridge). 2015;142(24):4374–84. doi: 10.1242/dev.129635 26552885 PMC4689222

[58] Daste F, Walrant A, Holst MR, Gadsby JR, Mason J, Lee JE, et al. Control of actin polymerization via the coincidence of phosphoinositides and high membrane curvature. Journal of Cell Biology. 2017;216(11):3745–65. doi: 10.1083/jcb.201704061 28923975 PMC5674896

[59] Herz J, Hamann U, Rogne S, Myklebost O, Gausepohl H, Stanley KK. Surface location and high affinity for calcium of a 500-kd liver membrane protein closely related to the LDL-receptor suggest a physiological role as lipoprotein receptor. EMBO J. 1988;7(13):4119–27. doi: 10.1002/j.1460-2075.1988.tb03306.x 3266596 PMC455121

[60] Herz J, Bock HH. Lipoprotein receptors in the nervous system. Annu Rev Biochem. 2002;71:405–34. doi: 10.1146/annurev.biochem.71.110601.135342 12045102

[61] Robinson MS. Adaptable adaptors for coated vesicles. Trends Cell Biol. 2004;14(4):167–74. doi: 10.1016/j.tcb.2004.02.002 15066634

[62] Lillis AP, Van Duyn LB, Murphy-Ullrich JE, Strickland DK. LDL receptor-related protein 1: Unique tissue-specific functions revealed by selective gene knockout studies. Vol. 88, Physiological Reviews. 2008. p. 887–918. doi: 10.1152/physrev.00033.2007 18626063 PMC2744109

[63] Nykjaer A, Willnow TE. The low-density lipoprotein receptor gene family: A cellular Swiss army knife? Trends Cell Biol. 2002;12(6):273–80. doi: 10.1016/s0962-8924(02)02282-1 12074887

[64] Willnow TE. The low-density lipoprotein receptor gene family: multiple roles in lipid metabolism. J Mol Med. 1999 Feb 12;77(3):306–15. doi: 10.1007/s001090050356 10090593

[65] Gumienny TL, Savage-Dunn C. TGF-β signaling in C. elegans. WormBook. 2013;1–34. 10.1895/wormbook.1.22.2 PMC5081272 23908056

[66] Gleason RJ, Akintobi AM, Grant BD, Padgett RW. BMP signaling requires retromer-dependent recycling of the type I receptor. Proc Natl Acad Sci U S A. 2014;111(7):2578–83. doi: 10.1073/pnas.1319947111 24550286 PMC3932876

[67] Bai Z, Grant BD. A TOCA/CDC-42/PAR/WAVE functional module required for retrograde endocytic recycling. Proc Natl Acad Sci U S A. 2015;112(12):E1443–52. doi: 10.1073/pnas.1418651112 25775511 PMC4378436

[68] Harterink M, Port F, Lorenowicz MJ, McGough IJ, Silhankova M, Betist MC, et al. A SNX3-dependent retromer pathway mediates retrograde transport of the Wnt sorting receptor Wntless and is required for Wnt secretion. Nat Cell Biol. 2011 Aug 3;13(8):914–23. doi: 10.1038/ncb2281 21725319 PMC4052212

[69] Reaves B, Horn M, Banting G. TGN38/41 recycles between the cell surface and the TGN: Brefeldin A affects its rate of return to the TGN. Mol Biol Cell. 1993;4(1):93–105. doi: 10.1091/mbc.4.1.93 8443412 PMC300903

[70] Belenkaya TY, Wu Y, Tang X, Zhou B, Cheng L, Sharma Y V., et al. The Retromer Complex Influences Wnt Secretion by Recycling Wntless from Endosomes to the Trans-Golgi Network. Dev Cell. 2008;14(1):120–31. doi: 10.1016/j.devcel.2007.12.003 18160348

[71] Yang PT, Lorenowicz MJ, Silhankova M, Coudreuse DYM, Betist MC, Korswagen HC. Wnt Signaling Requires Retromer-Dependent Recycling of MIG-14/Wntless in Wnt-Producing Cells. Dev Cell. 2008;14(1):140–7. doi: 10.1016/j.devcel.2007.12.004 18160347

[72] Sawa H, Korswagen HC. Wnt signaling in C. elegans *. 2013;1–30. 10.1895/wormbook.1.7.2 PMC5402212 25263666

[73] Roquemore EP, Banting G. Efficient trafficking of TGN38 from the endosome to the trans-Golgi network requires a free hydroxyl group at position 331 in the cytosolic domain. Mol Biol Cell. 1998;9(8):2125–44. doi: 10.1091/mbc.9.8.2125 9693371 PMC25467

[74] Humphrey JS, Peters PJ, Yuan LC, Bonifacino JS. Localization of TGN38 to the trans-Golgi network: involvement of a cytoplasmic tyrosine-containing sequence. Journal of Cell Biology. 1993 Mar 1;120(5):1123–35. doi: 10.1083/jcb.120.5.1123 8436587 PMC2119736

[75] Sun T, Wang X, Lu Q, Ren H, Zhang H. CUP-5, the C. elegans ortholog of the mammalian lysosomal channel protein MLN1/TRPML1, is required for proteolytic degradation in autolysosomes. Autophagy. 2011;7(11):1308–15. doi: 10.4161/auto.7.11.17759 21997367

[76] Campbell EM, Fares H. Roles of CUP-5, the Caenorhabditis elegans orthologue of human TRPML1, in lysosome and gut granule biogenesis. BMC Cell Biol. 2010 Dec 11;11(1):40. doi: 10.1186/1471-2121-11-40 20540742 PMC2891664

[77] Fares H, Greenwald I. Regulation of endocytosis by CUP-5, the Caenorhabditis elegans mucolipin-1 homolog. Nat Genet. 2001 May;28(1):64–8. doi: 10.1038/ng0501-64 11326278

[78] Arighi CN, Harmell LM, Aguilar RC, Haft CR, Bonifacino JS. Role of the mammalian retromer in sorting of the cation-independent mannose 6-phosphate receptor. Journal of Cell Biology. 2004;165(1):123–33. doi: 10.1083/jcb.200312055 15078903 PMC2172094

[79] Mu FT, Callaghan JM, Steele-Mortimer O, Stenmark H, Parton RG, Campbell PL, et al. EEA1, an early endosome-associated protein. EEA1 is a conserved α- helical peripheral membrane protein flanked by cysteine “fingers” and contains a calmodulin-binding IQ motif. Journal of Biological Chemistry. 1995;270(22):13503–11. 7768953 10.1074/jbc.270.22.13503

[80] Patki V, Virbasius J, Lane WS, Toh BH, Shpetner HS, Corvera S. Identification of an early endosomal protein regulated by phosphatidylinositol 3-kinase. Proc Natl Acad Sci U S A. 1997;94(14):7326–30. doi: 10.1073/pnas.94.14.7326 9207090 PMC23820

[81] Gaullier JM, Simonsen A, D’Arrigo A, Bremnes B, Stenmark H, Aasland R. FYVE fingers bind PtdIns(3)P. Nature. 1998 Jul;394(6692):432–3. doi: 10.1038/28767 9697764

[82] Sharma M, Srinivas Panapakkam Giridharan S, Rahajeng J, Caplan S, Naslavsky N. MICAL-L1. Commun Integr Biol. 2010 Mar 28;3(2):181–3. 20585517 10.4161/cib.3.2.10845 PMC2889981

[83] Rahajeng J, Panapakkam Giridharan SS, Cai B, Naslavsky N, Caplan S. MICAL-L1 is a tubular endosomal membrane hub that connects Rab35 and Arf6 with Rab8a. Traffic. 2012;13(1):82–93. doi: 10.1111/j.1600-0854.2011.01294.x 21951725 PMC3302426

[84] Giridharan SSP, Cai B, Vitale N, Naslavsky N, Caplan S. Cooperation of MICAL-L1, syndapin2, and phosphatidic acid in tubular recycling endosome biogenesis. Mol Biol Cell. 2013;24(11):1776–90. doi: 10.1091/mbc.E13-01-0026 23596323 PMC3667729

[85] Cai B, Xie S, Caplan S, Naslavsky N. GRAF1 forms a complex with MICAL-L1 and EHD1 to cooperate in tubular recycling endosome vesiculation. Front Cell Dev Biol. 2014;2(MAY):1–14. doi: 10.3389/fcell.2014.00022 25364729 PMC4214196

[86] Savage-Dunn C, Padgett RW. The TGF-β family in Caenorhabditis elegans. Cold Spring Harb Perspect Biol. 2017 Jun 1;9(6). 10.1101/cshperspect.a022178 PMC5453393 28096268

[87] Liu Z, Shi H, Nzessi AK, Norris A, Grant BD, Liu J. Tetraspanins TSP-12 and TSP-14 function redundantly to regulate the trafficking of the type II BMP receptor in Caenorhabditis elegans. Proceedings of the National Academy of Sciences [Internet]. 2020 Feb 11 [cited 2023 Mar 29];117(6):2968–77. Available from: https://pnas.org/doi/full/10.1073/pnas.1918807117 10.1073/pnas.1918807117 PMC7022192 31988138

[88] Yoshida S, Morita K, Mochii M, Ueno N. Hypodermal expression of Caenorhabditis elegans TGF-β type I receptor SMA-6 is essential for the growth and maintenance of body length. Dev Biol. 2001 Dec 1;240(1):32–45. 11784045 10.1006/dbio.2001.0443

[89] Sato M, Sato K, Fonarev P, Huang CJ, Liou W, Grant BD. Caenorhabditis elegans RME-6 is a novel regulator of RAB-5 at the clathrin-coated pit. Nat Cell Biol. 2005;7(6):559–69. doi: 10.1038/ncb1261 15895077 PMC1398054

[90] Chavrier P, Parton RG, Hauri HP, Simons K, Zerial M. Localization of low molecular weight GTP binding proteins to exocytic and endocytic compartments. Cell. 1990;62(2):317–29. doi: 10.1016/0092-8674(90)90369-p 2115402

[91] Mooren OL, Galletta BJ, Cooper JA. Roles for actin assembly in endocytosis. Annu Rev Biochem. 2012;81:661–86. doi: 10.1146/annurev-biochem-060910-094416 22663081

[92] Kaksonen M, Sun Y, Drubin DG. A pathway for association of receptors, adaptors, and actin during endocytic internalization. Cell. 2003;115(4):475–87. doi: 10.1016/s0092-8674(03)00883-3 14622601

[93] Yarar D, Waterman-Storer CM, Schmid SL. A dynamic actin cytoskeleton functions at multiple stages of clathrin-mediated endocytosis. Mol Biol Cell. 2005;16(2):964–75. doi: 10.1091/mbc.e04-09-0774 15601897 PMC545926

[94] Munn AL. Molecular requirements for the internalisation step of endocytosis: Insights from yeast. Biochim Biophys Acta Mol Basis Dis. 2001;1535(3):236–57. doi: 10.1016/s0925-4439(01)00028-x 11278164

[95] Kaksonen M, Toret CP, Drubin DG. Harnessing actin dynamics for clathrin-mediated endocytosis. Nat Rev Mol Cell Biol. 2006;7(6):404–14. doi: 10.1038/nrm1940 16723976

[96] Derivery E, Sousa C, Gautier JJ, Lombard B, Loew D, Gautreau A. The Arp2/3 activator WASH controls the fission of endosomes through a large multiprotein complex. Dev Cell. 2009;17(5):712–23. doi: 10.1016/j.devcel.2009.09.010 19922875

[97] Gomez TS, Billadeau DD. A FAM21-containing WASH complex regulates retromer-dependent sorting. Dev Cell. 2009 Nov 1;17(5):699–711. doi: 10.1016/j.devcel.2009.09.009 19922874 PMC2803077

[98] Chakrabarti R, Lee M, Higgs HN. Multiple roles for actin in secretory and endocytic pathways. Current Biology. 2021;31(10):R603–18. doi: 10.1016/j.cub.2021.03.038 34033793 PMC9759210

[99] Pelkmans L, Fava E, Grabner H, Hannus M, Habermann B, Krausz E, et al. Genome-wide analysis of human kinases in clathrin- and caveolae/raft- mediated endocytosis. Nature. 2005;436(7047):78–86. doi: 10.1038/nature03571 15889048

[100] Govindaraghavan M, McGuire Anglin SL, Shen KF, Shukla N, De Souza CP, Osmani SA. Identification of interphase functions for the NIMA kinase involving M`icrotubules and the ESCRT Pathway. Heitman J, editor. PLoS Genet. 2014 Mar 27;10(3):e1004248. doi: 10.1371/journal.pgen.1004248 24675878 PMC3967960

[101] Collinet C, Stöter M, Bradshaw CR, Samusik N, Rink JC, Kenski D, et al. Systems survey of endocytosis by multiparametric image analysis. Nature. 2010;464(7286):243–9. doi: 10.1038/nature08779 20190736

[102] Vaz Meirelles G, Ferreira Lanza DC, Da Silva JC, Santana Bernachi J, Paes Leme AF, Kobarg J. Characterization of hNek6 interactome reveals an important role for its short N-terminal domain and colocalization with proteins at the centrosome. J Proteome Res. 2010;9(12):6298–316. doi: 10.1021/pr100562w 20873783

[103] De Souza EE, Meirelles GV, Godoy BB, Perez AM, Smetana JHC, Doxsey SJ, et al. Characterization of the human NEK7 interactome suggests catalytic and regulatory properties distinct from those of NEK6. J Proteome Res. 2014;13(9):4074–90. doi: 10.1021/pr500437x 25093993 PMC4156247

[104] Meirelles GV, Perez AM, de Souza EE, Basei FL, Papa PF, Melo Hanchuk TD, et al. “Stop Ne(c)king around”: How interactomics contributes to functionally characterize Nek family kinases. World J Biol Chem. 2014;5(2):141–60. doi: 10.4331/wjbc.v5.i2.141 24921005 PMC4050109

[105] Kumar G, Thomas B, Mensa-Wilmot K. Pseudokinase NRP1 facilitates endocytosis of transferrin in the African trypanosome. Sci Rep. 2022;12(1):1–15. 36329148 10.1038/s41598-022-22054-x PMC9633767

[106] Zhang Y, Wang X. Targeting the Wnt/β-catenin signaling pathway in cancer. J Hematol Oncol. 2020;13(1):1–16. 33276800 10.1186/s13045-020-00990-3 PMC7716495

[107] Bach DH, Park HJ, Lee SK. The Dual Role of Bone Morphogenetic Proteins in Cancer. Mol Ther Oncolytics. 2018;8(March):1–13. doi: 10.1016/j.omto.2017.10.002 29234727 PMC5723373

[108] Wakefield LM, Hill CS. Beyond TGFβ: Roles of other TGFβ superfamily members in cancer. Nat Rev Cancer. 2013;13(5):328–41. 23612460 10.1038/nrc3500 PMC7608560

[109] Schmid SL. Reciprocal regulation of signaling and endocytosis: Implications for the evolving cancer cell. Journal of Cell Biology. 2017;216(9):2623–32. doi: 10.1083/jcb.201705017 28674108 PMC5584184

[110] Mosesson Y, Mills GB, Yarden Y. Derailed endocytosis: An emerging feature of cancer. Nat Rev Cancer. 2008;8(11):835–50. doi: 10.1038/nrc2521 18948996

[111] Mellman I, Yarden Y. Endocytosis and cancer. Cold Spring Harb Perspect Biol. 2013;5(12). doi: 10.1101/cshperspect.a016949 24296170 PMC3839607

[112] Chen PH, Bendris N, Hsiao YJ, Reis CR, Mettlen M, Chen HY, et al. Crosstalk between CLCb/Dyn1-mediated adaptive clathrin-mediated endocytosis and epidermal growth factor receptor signaling increases metastasis. Dev Cell. 2017 Feb 6;40(3):278–288.e5. doi: 10.1016/j.devcel.2017.01.007 28171750 PMC5740869

[113] Zhou L, Wang Z, Xu X, Wan Y, Qu K, Fan H, et al. Nek7 is overexpressed in hepatocellular carcinoma and promotes hepatocellular carcinoma cell proliferation in vitro and in vivo. Oncotarget. 2016 Apr 5;7(14):18620–30. doi: 10.18632/oncotarget.7620 26921196 PMC4951314

[114] Yan Z, Da Q, Li Z, Lin Q, Yi J, Su Y, et al. Inhibition of NEK7 suppressed hepatocellular carcinoma progression by mediating cancer cell pyroptosis. Front Oncol. 2022 Feb 10;12(February):812655. doi: 10.3389/fonc.2022.812655 35223495 PMC8866693

[115] Nassirpour R, Shao L, Flanagan P, Abrams T, Jallal B, Smeal T, et al. Nek6 mediates human cancer cell transformation and is a potential cancer therapeutic target. Molecular Cancer Research. 2010;8(5):717–28. doi: 10.1158/1541-7786.MCR-09-0291 20407017

[116] De Donato M, Righino B, Filippetti F, Battaglia A, Petrillo M, Pirolli D, et al. Identification and antitumor activity of a novel inhibitor of the NIMA-related kinase NEK6. Sci Rep. 2018;8(1):1–13. 30375481 10.1038/s41598-018-34471-y PMC6207720

[117] De Donato M, Fanelli M, Mariani M, Raspaglio G, Pandya D, He S, et al. Nek6 and Hif-1α cooperate with the cytoskeletal gateway of drug resistance to drive outcome in serous ovarian cancer. Am J Cancer Res. 2015;5(6):1862–77. 26269749 PMC4529609

[118] Stiernagle T. Maintenance of C. elegans. WormBook. 2006;(1999):1–11. doi: 10.1895/wormbook.1.101.1 18050451 PMC4781397

[119] Ghanta KS, Ishidate T, Mello CC. Microinjection for precision genome editing in Caenorhabditis elegans. STAR Protoc. 2021;2(3):100748. doi: 10.1016/j.xpro.2021.100748 34505086 PMC8417391

[120] Philipp TM, Gong W, Köhnlein K, Ohse VA, Müller FI, Priebs J, et al. SEMO-1, a novel methanethiol oxidase in Caenorhabditis elegans, is a pro-aging factor conferring selective stress resistance. BioFactors. 2022;48(3):699–706. doi: 10.1002/biof.1836 35316559

[121] Frøkjær-Jensen C, Wayne Davis M, Hopkins CE, Newman BJ, Thummel JM, Olesen SP, et al. Single-copy insertion of transgenes in Caenorhabditis elegans. Nat Genet. 2008;40(11):1375–83. doi: 10.1038/ng.248 18953339 PMC2749959

[122] Schindelin J, Arganda-Carreras I, Frise E, Kaynig V, Longair M, Pietzsch T, et al. Fiji: An open-source platform for biological-image analysis. Nat Methods. 2012;9(7):676–82. doi: 10.1038/nmeth.2019 22743772 PMC3855844

