# Supplementary figures and images for: Conserved NIMA kinases regulate multiple steps of endocytic trafficking
Source: PLoS Genet. 2023 Apr 26;19(4):e1010741. doi: 10.1371/journal.pgen.1010741 (PMC10166553; doi:10.1371/journal.pgen.1010741)

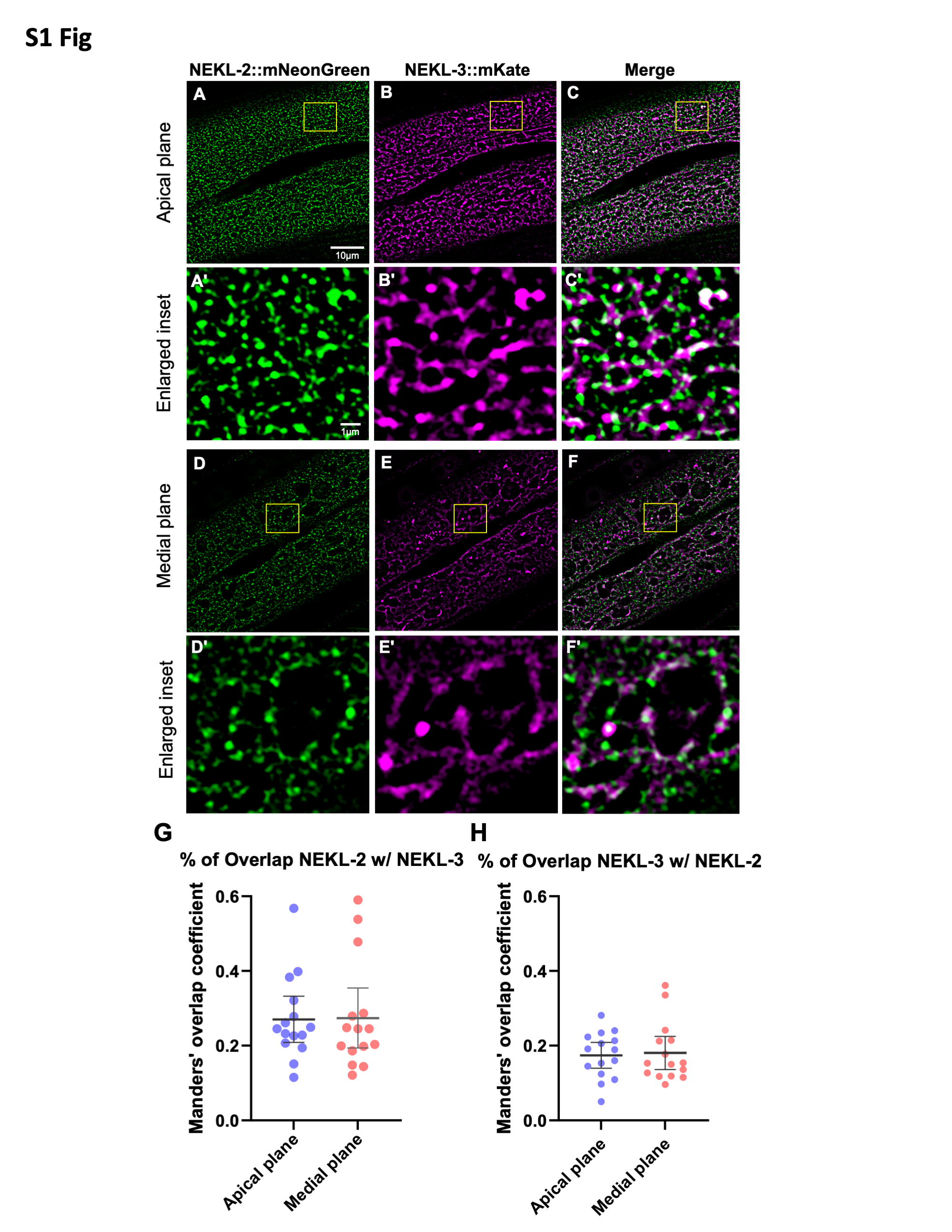

Supplement: S1 Fig — Colocalization assays were carried out in adult worms expressing both NEKL-2::mNeonGreen and NEKL-3::mKate. (A–C, A’–C’, D–F, and D’–F’) Representative images of the apical region of hyp7 in adult worms expressing NEKL-2::mNeonGreen and NEKL-3::mKate (A–C and A’–C’) as well as the medial plane of hyp7 (D–F and D’–F’). Here the medial plane is considered the plane ~1 μm below the apical surface. Scale bar in A = 10 μm for A–F. Scale bar in A’ = 1 μm for A’–F’. (G, H) Manders’ coefficient was calculated and plotted for individual worms in nekl-2::mNeonGreen; nekl-3::mKate strains. The fraction of NEKL-2::mNeonGreen puncta overlapping with NEKL-3::mKate puncta in the apical and medial plane (G) and vice versa (H) are shown. Raw data are available in S1 File. (TIFF) [file pgen.1010741.s001.tiff]

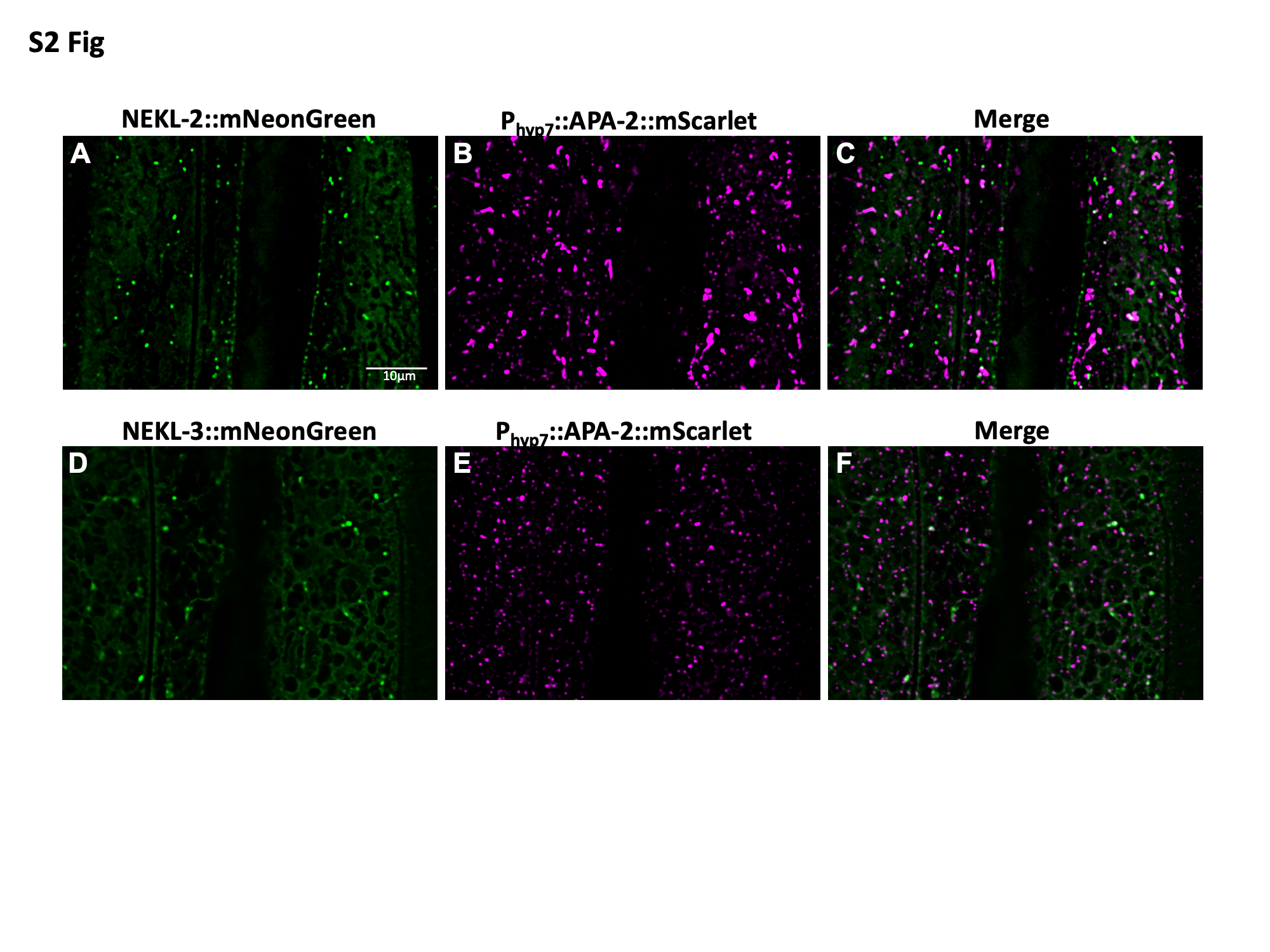

Supplement: S2 Fig — (A–F) Colocalization assays were performed on strains expressing either NEKL-2::mNeonGreen (A–C) or NEKL-3::mNeonGreen (D–F) with APA-2::mScarlet, the alpha subunit of the AP2 adaptor complex, which is present in clathrin-coated pits. Note that these images were collected on a different confocal microscope and thus appear somewhat different than images in the paper (see Materials and Methods). Scale bar in A = 10 μm in A–F. (TIFF) [file pgen.1010741.s002.tiff]

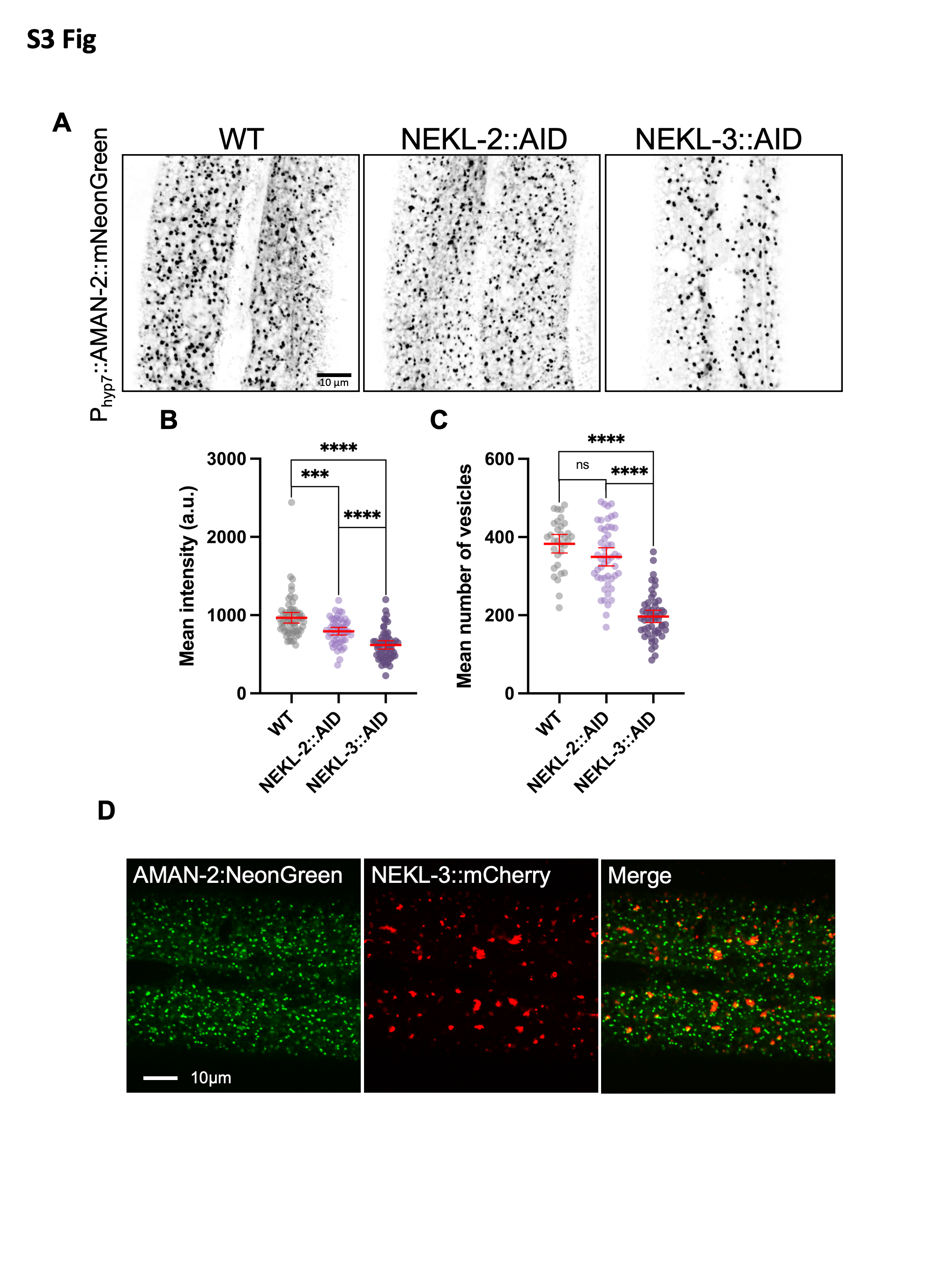

Supplement: S3 Fig — (A) Representative confocal images of Phyp7::AMAN-2::mNeonGreen expression in the indicated backgrounds. (B, C) Mean intensity of Phyp7::AMAN-2::mNeonGreen expression (B) and the mean number of Phyp7::AMAN-2::mNeonGreen-positive vesicles (C) for individual worms were plotted in the graphs. (D) Representative confocal images of Phyp7::AMAN-2::mNeonGreen co-expressed with a functional multi-copy NEKL-3::mCherry reporter. Error bars represent the 95% confidence intervals. p-Values were obtained by comparing means using an unpaired t-test: ****p < 0.0001, ***p < 0.001; ns, not significant (p > 0.05). Raw data are available in S1 File. (TIFF) [file pgen.1010741.s003.tiff]

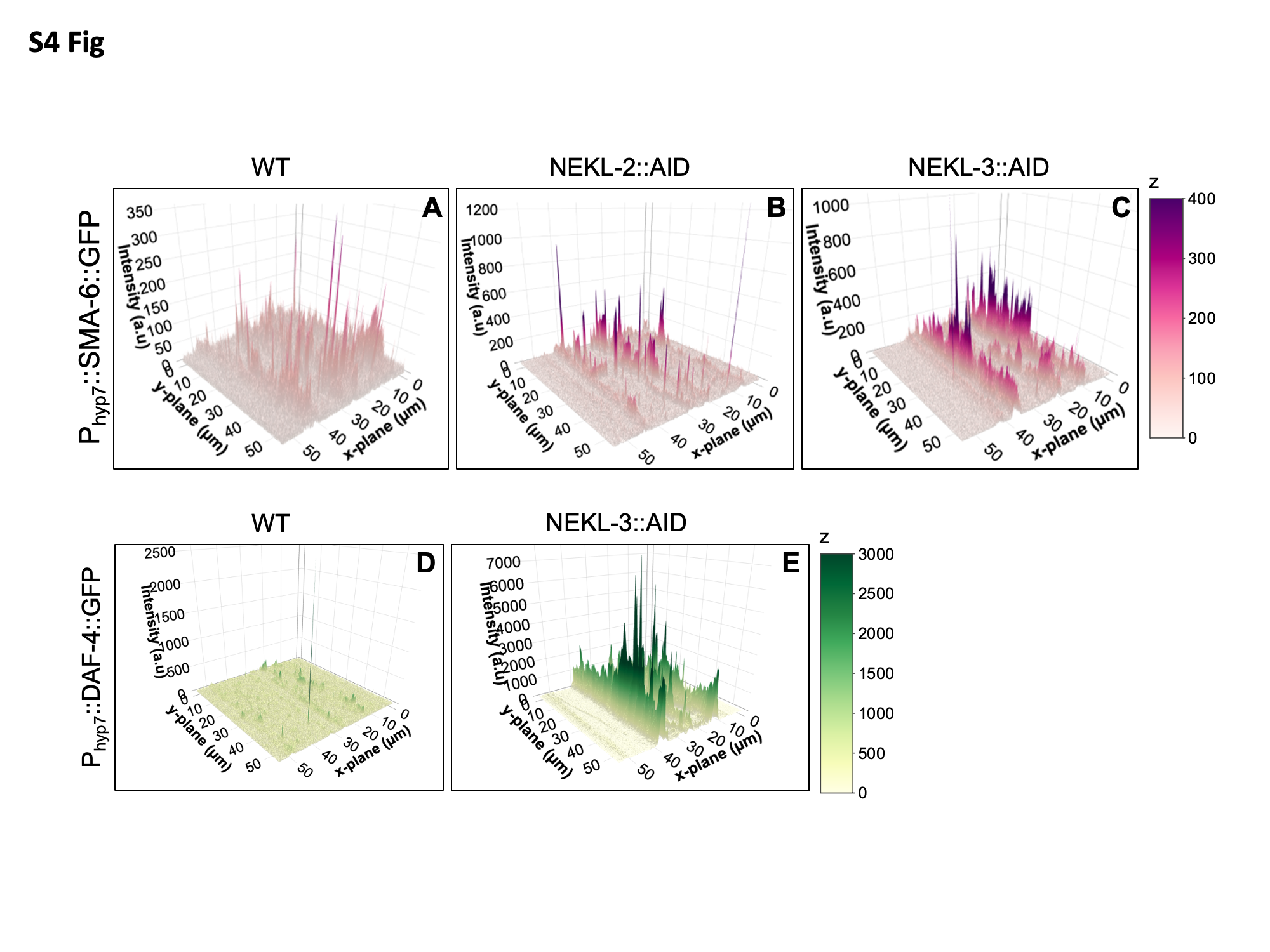

Supplement: S4 Fig — (A–C) Three-dimensional plots showing the individual pixel intensity along the x and y planes for the fluorescent images presented in Fig 3C–3E. (D,E) Three-dimensional plots showing the pixel intensity value along the x and y planes for the fluorescent images presented in Fig 3F and 3G. We note that the basal boundary occurs in the x axes at approximately 40 μm and 10 μm whereas the seam cell boundary occurs between 20–30 μm. (TIFF) [file pgen.1010741.s004.tiff]

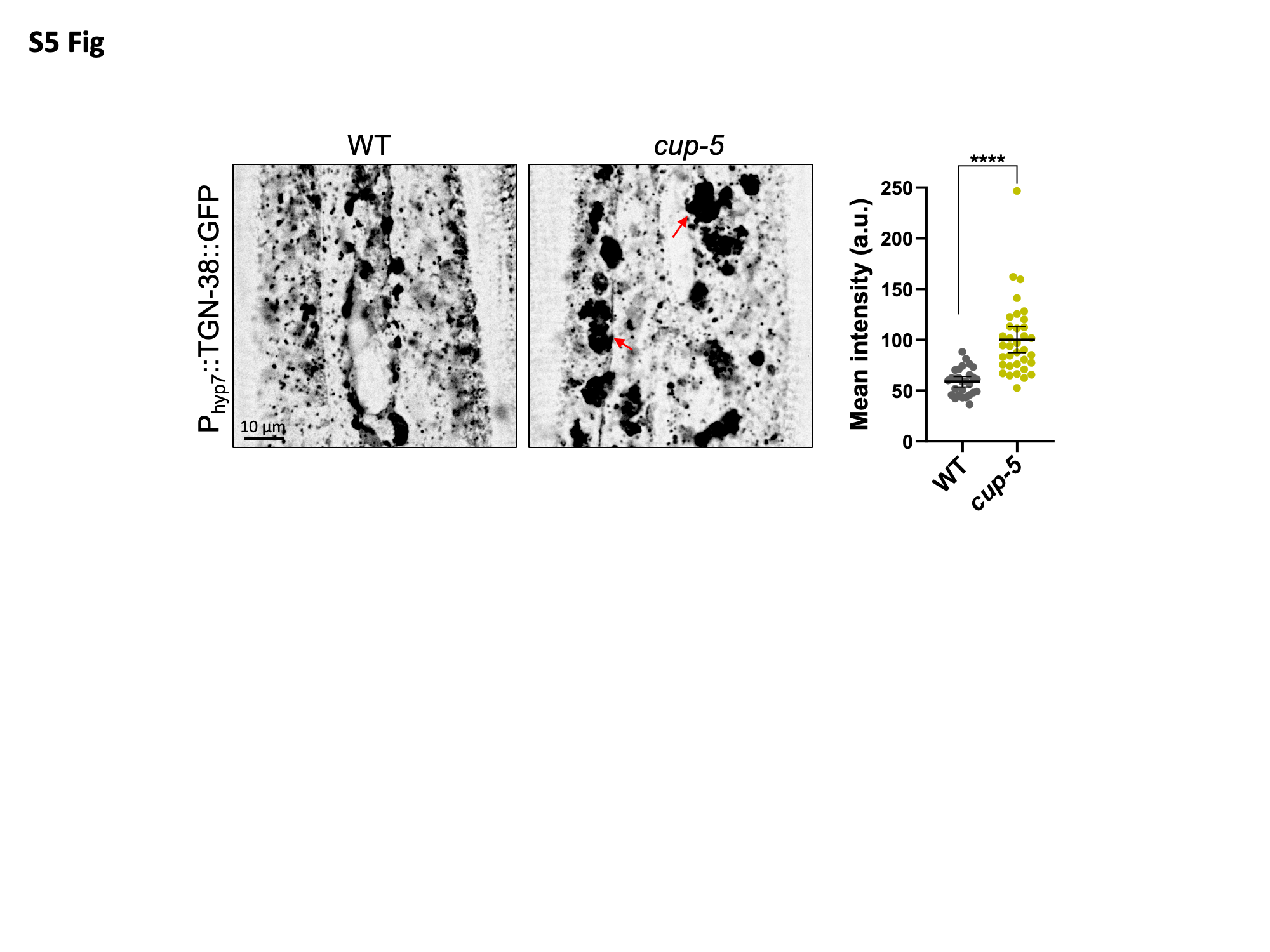

Supplement: S5 Fig — Representative images of Phyp-7::TGN-38::GFP expression in auxin-treated day-2 adults in wildtype and cup-5 mutant background. Red arrows indicate accumulation of cargoes in vesicle-like structures. Mean intensity values of the Phyp-7::TGN-38::GFP expression for individual worms are plotted in the graph. Error bars represent the 95% confidence intervals. p-Values were obtained by comparing means using an unpaired t-test: ****p < 0.0001, ns, not significant (p > 0.05). Raw data are available in S1 File. (TIFF) [file pgen.1010741.s005.tiff]

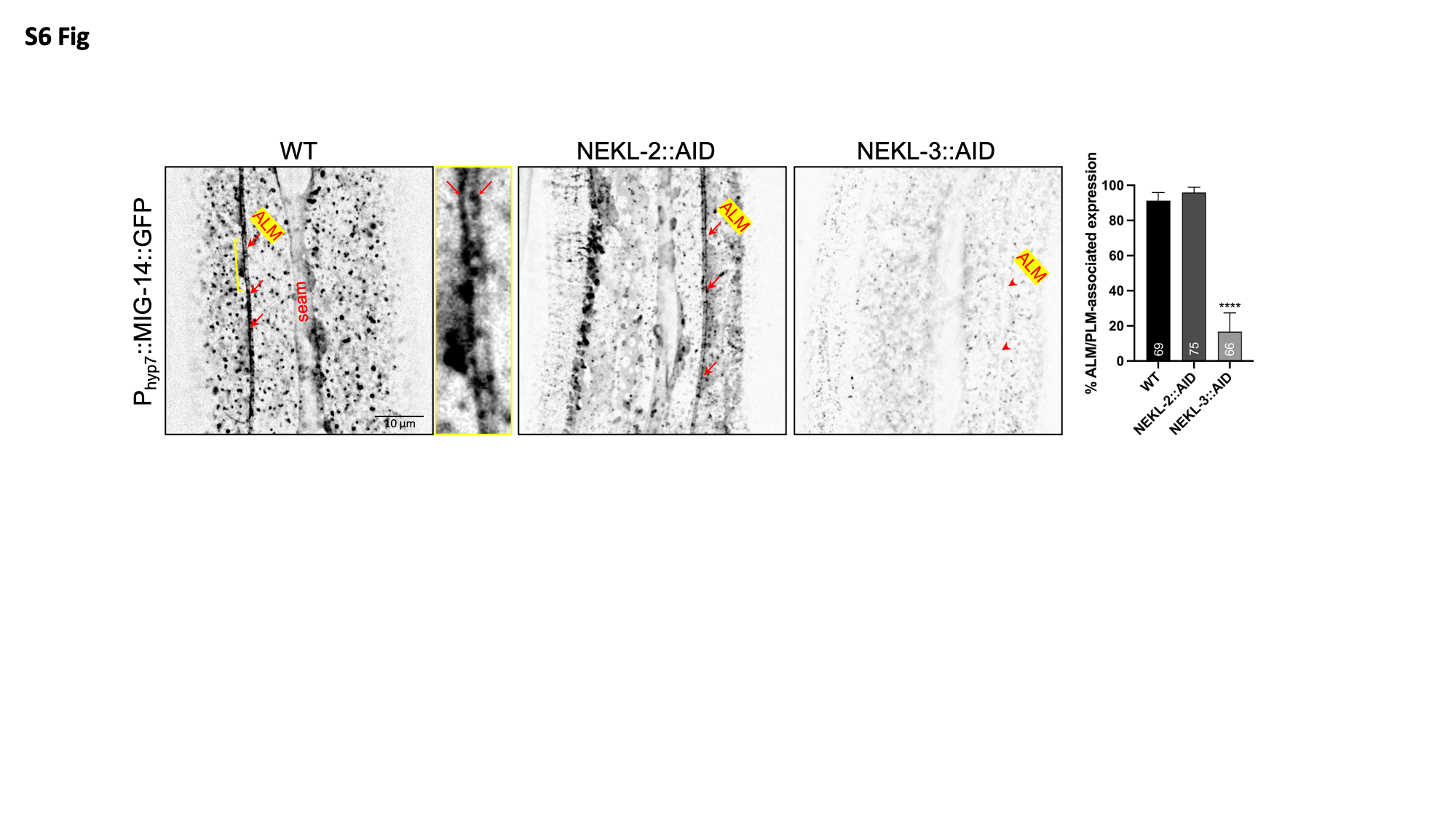

Supplement: S6 Fig — Representative images of Phyp-7::MIG-14::GFP expression in auxin-treated wild-type, nekl-2::aid, and nekl-3::aid day-2 adults in the apical/medial plane. Red arrows show the presence of MIG-14::GFP expression in wild-type and nekl-2::aid adults at the hyp7 membrane surrounding the ALM neuron. The red arrowhead indicates the absence of MIG-14::GFP expression in nekl-3::aid worms. Scale bar = 10 μm in the three lower-magnification images. The yellow bracket along the ALM neuron in wild type indicates the region show in the higher magnification image (yellow outline). The bar graph shows the percentage of worms exhibiting Phyp-7::MIG-14::GFP expression surrounding the ALM/PLM neurons in the indicated backgrounds. Numbers of worms are indicated for each bar. Error bars represent the 95% confidence interval. p-Values were obtained using Fisher’s exact test: ****p < 0.0001. Raw data are available in S1 File. (TIFF) [file pgen.1010741.s006.tiff]

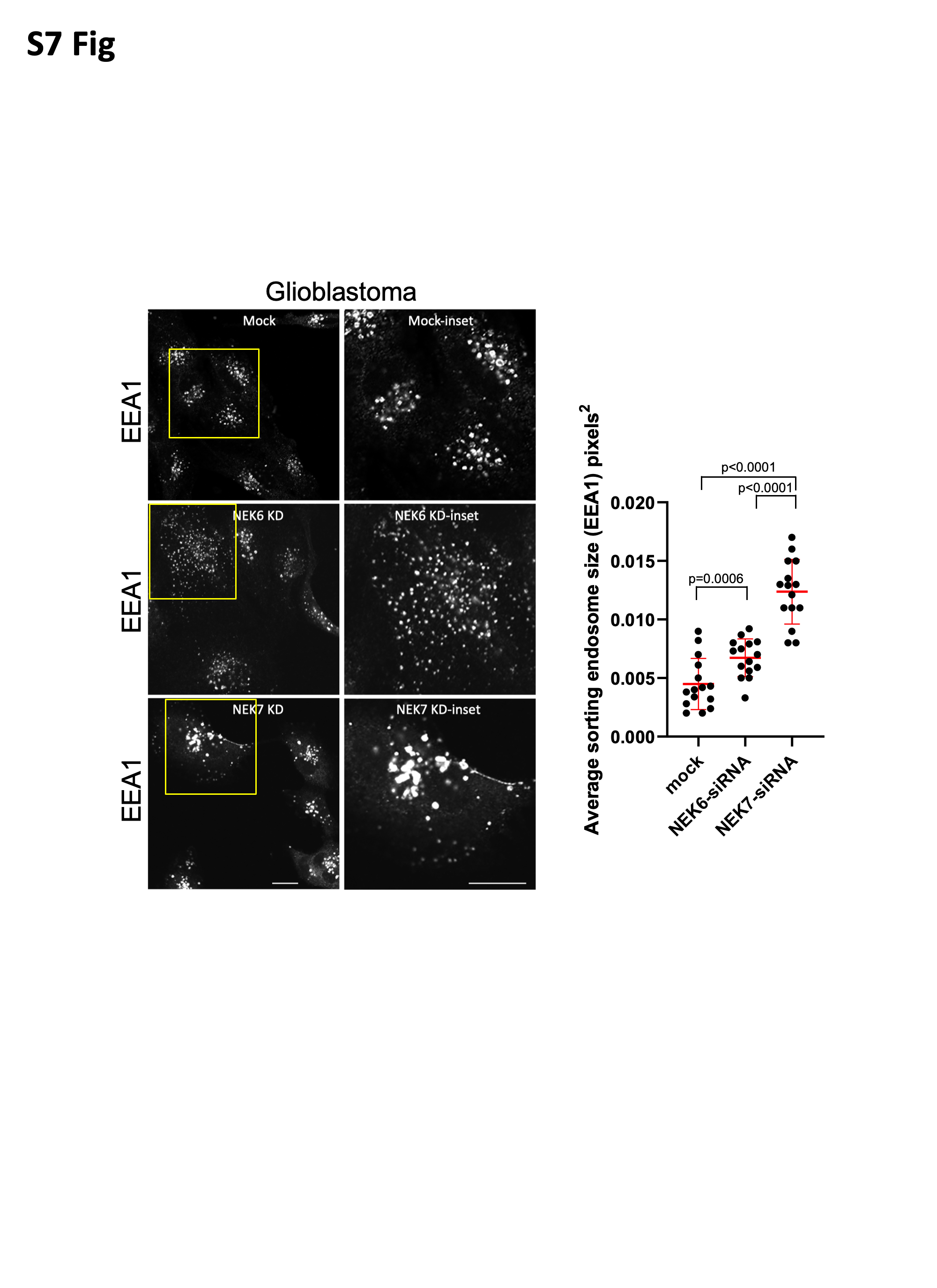

Supplement: S7 Fig — Mock-transfected cells, NEK6 siRNA-transfected cells, or NEK7 siRNA-transfected cells were plated on coverslips and immunostained with antibodies against the sorting endosome marker protein EEA1. Average sizes of early/sorting endosomes are shown for the three conditions, with significant differences observed between the mock-transfected cells and both siRNA treatments as well as between NEK6 siRNA and NEK7 siRNA-transfected cells. Yellow box in panels indicates region of enlarged inset. Scale bars = 10 μm. (TIFF) [file pgen.1010741.s007.tiff]

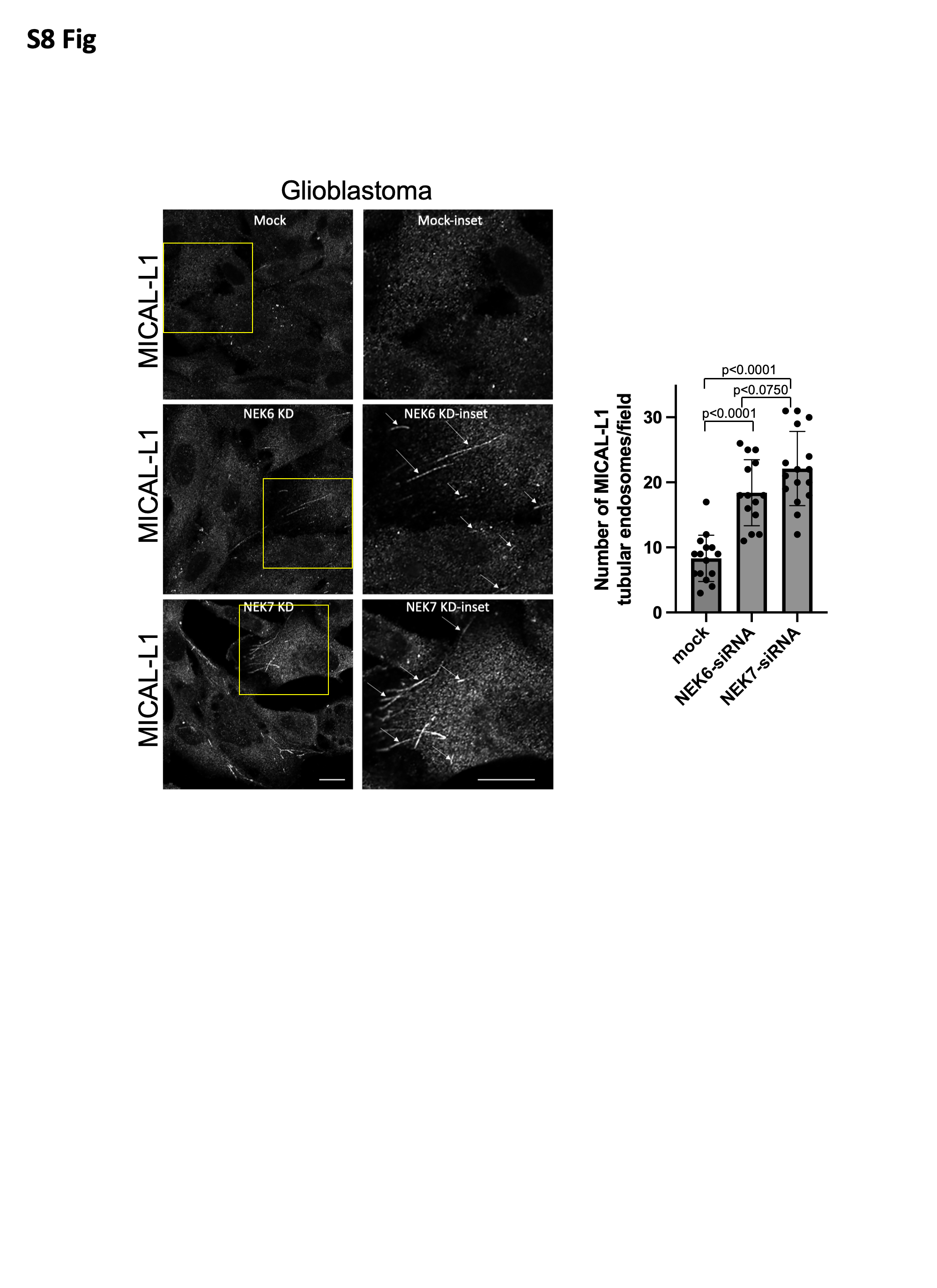

Supplement: S8 Fig — Glioblastoma cells were mock transfected or were transfected with NEK6 siRNA or NEK7 siRNA. Transfected cells were then plated on coverslips and immunostained with antibodies against the tubular recycling endosome marker protein MICAL-L1. Average numbers of MICAL-L1 tubular endosomes per field were determined for the three conditions, with significant differences observed between the mock-transfected cells and both siRNA treatments. Yellow box in panels indicates region of enlarged inset. Scale bars = 10 μm. (TIFF) [file pgen.1010741.s008.tiff]
