# Supplementary material for: Conserved NIMA kinases regulate multiple steps of endocytic trafficking
Source: PLoS Genet. 2023 Apr 26;19(4):e1010741. doi: 10.1371/journal.pgen.1010741 (PMC10166553; doi:10.1371/journal.pgen.1010741)
Supplement: S1 Table — (PDF) [file pgen.1010741.s009.pdf]

S1 Table. List of strains used in this study

Strain	Genotype
PHX1714	*syb1714 (rab-11.1) mScarlet::rab-11.1*
RT3533	*pwSi7[phyp7::GFP::rab-7]*
RT3596	*pw27[nekl-2::aid]; ieSi57[peft-3::mRuby::tir1]; pwSi7[phyp7::GFP::rab-7]*
RT3597	*pw29[nekl-3::aid]; ieSi57[peft-3::mRuby::tir1]; pwSi7[phyp7::GFP::rab-7]*
RT3649	*pw27[nekl-3::aid]; ieSi57[peft-3::mRuby::tir1]; pwls439[GFP::rab-5]*
RT3650	*pw29[nekl-3::aid]; ieSi57[peft-3::mRuby::tir1]; pwls439[GFP::rab-5]*
RT3995	*bzls166[pmec4::mCherry]; pwSi202[phyp7::aman-2::mNeonGreen]*
RT4000	*pwSi207[phyp7::sma-6::GFP]*
RT4266	*pwSi363[phyp7::mig-14::GFP]*
RT4271	*pwSi368[phyp7::daf-4::GFP]*
RT4337	*pw43[tgn-38::gfp]*
WY1193	*nekl-2(fd100)[nekl-2::mNeonGreen::3xFlag]); nekl-3(fd106)[nekl-3:: mKate2::3xFlag]*
WY1716	*pw29[nekl-3::aid]; ie57[peft-3::mRuby::tir-1]; pwSi207[phyp7::sma-6::GFP]*
WY1717	*pw29[nekl-3::aid]; ie57[peft-3::mRuby::tir-1]; pwSi363[phyp7::mig-14::GFP]*
WY1719	*pw29[nekl-3::aid]; ie57[peft-3::mRuby::tir-1]; pwSi368[phyp7::daf-4::GFP]*
WY1728	*pw27[nekl-2::aid]; ie57[peft-3::mRuby::tir-1]; pwSi207[phyp7::sma-6::GFP]*
WY1736	*pw27[nekl-2::aid]; ie57[peft-3::mRuby::tir-1]; pw43[tgn-38::GFP] line 1*
WY1741	*pw27[nekl-2::aid); ie57[peft-3::mRuby::tir-1]; pwSi363[phyp7::mig-14::GFP] line 1*
WY1758	*pw29[nekl-3::aid]; ie57[peft-3::mRuby::tir-1]; pw43[tgn-38::GFP] line 1*
WY1763	*pw1543[GFP::rab-5]*
WY1838	*syb1714 (mScarlet::rab-11.1); pw29[nekl-3::aid]; pwSi10[phyp-7::BFP::tir-1]*
WY1844	*syb1714 (mScarlet::rab-11.1); pw27[nekl-2::aid]; pwSi10[phyp-7::BFP::tir-1]*
WY1917	*pw27[nekl-2::aid]; ie57[peft-3::mRuby::tir-1]; pwSi202[phyp7::aman-2::mNeonGreen]*
WY1935	*pw29[nekl-3::aid]; ie57[peft-3::mRuby::tir-1]; pwSi202[phyp7::aman-2::mNeonGreen]*
WY1941	*nekl-3::mKate CRISPR (fd106); pwls439[GFP::rab-5]*
WY1943	*nekl-3::mKate CRISPR (fd106); pwSi7[phyp7::GFP::rab-7]*
WY1944	*nekl-2::mKate CRISPR (fd127); pwls439[GFP::rab-5]*
WY1945	*nekl-2::mKate CRISPR (fd127); pwSi7[phyp7::GFP::rab-7]*
WY1998	*pw29[nekl-3::aid); ie57[peft-3::mRuby::tir-1]; pw43[tgn-38::GFP]; cup-5(fd395)*
WY2000	*pw27[nekl-2::aid); ie57[peft-3::mRuby::tir-1]; pw43[tgn-38::GFP]; cup-5 (fd397)*
WY1464	*nekl-2::mNeonGreen CRISPR (fd100); pwSi36[phyp7::mScarlet::apa-2]*
WY1469	*nekl-3::mNeonGreen CRISPR (fd118); pwSi36[phyp7::mScarlet::apa-2]*
