# Supplementary material for: Conserved NIMA kinases regulate multiple steps of endocytic trafficking
Source: PLoS Genet. 2023 Apr 26;19(4):e1010741. doi: 10.1371/journal.pgen.1010741 (PMC10166553; doi:10.1371/journal.pgen.1010741)
Supplement: Attachment — Submitted filename: Response to reviewers.pdf [file pgen.1010741.s011.pdf]

We thank the reviewers for their positive comments as well as their suggestions for improvements and future directions. This led us to carry out several additional requested experiments as well as several additional experiment not specifically requested. We address each point below.

Reviewer #1

The Fay lab previously demonstrated that the NIMA like kinases NEKL-2 and NEKL-3 localize to foci and regulate clathrin-mediated LRP-1 endocytosis at the apical membrane of the Hyp7 syncytium. Here Joseph et al further characterize the role NEKL-2 and NEKL-3 in endosome trafficking. They demonstrate that NEKL-3 colocalizes with RAB-5 and RAB-7 positive endosomes while NEKL-2 shows some colocalization with RAB-5 and much less with RAB-7. NEKL-2 and NEKL-3 do not grossly colocalize indicating that they are mostly on distinct compartments. Auxin mediated depletion of NEKL-2 resulted in tubulation of RAB-5 compartments and depletion of NEKL-3 resulted in tubulation of RAB-11 compartments. The basal recycling cargo SMA-6 (clathrin dependent) and DAF-4 (clathrin independent) accumulate at or near the plasma membrane in NEKL-2 and NEKL-3 depleted animals consistent with their role in trafficking. However, intracellular cargo receptors MIG-14 and TGN-38 which originate from the Golgi are reduced in NEKL-2 and NEKL-3 depleted animals to different degrees suggesting they are being degraded in the lysosome. Consistent with that hypothesis TGN-38 levels are increased in NEKL-2 and NEKL-3 depleted animals when endosome to lysosome trafficking is impeded in a cup-5 mutant. Consistent with the NEKL-2 and NEKL-3 results, siRNA depletion of NEK6 and NEK7 (human NEKL-3 homologs) in HeLa cells results in decreased levels of Mannose-6-phosphate receptor suggesting that it too might be targeted to the lysosome. siRNA of NEK6 and NEK7 increased EEA1 early endosome size in HeLa and cells and siNEK7, but not siNEK6 does the same in Glioblastoma cells. siNEK6 and siNEK increase the area (length?) of MICAL-L1 tubules in both HeLa and glioblastoma cells. These data suggest that there is a conserved role for NIMA like kinases in endosome fission or fusion.

Strengths

This paper is well written and easy to read without notable typographical errors.
Most of the data is quantified and clearly displayed. All the image quantification data is available in an excel file.
The novelty of the study is that NIMA like kinases regulate endosome tubulation.
Experimentation in both C. elegans and human cell lines broadens the impact of the findings.
The experiments are well done.

Weaknesses

The study is a bit of a look and see approach rather than hypothesis driven. I find the tubulation phenotypes are the most dramatic and interesting as they point toward a specific function for the NEKL-2 and 3. I would have liked to see more experiments focused on the tubulation defects. A specific role for the NIMA kinases is not determined.

We agree and are currently taking several approaches that will hopefully lead to a clearer and more precise understanding of the molecular and cellular functions of NEKs/NEKLs in trafficking. Please stay tuned!

Supplementary data on glioblastoma cells is not quantified.

This has now been added. See new Fig S7 and Fig S8, corresponding legends, and materials and methods (lines 527–534).

Points of consideration

NEKL-3 appears to have a reticulate localization pattern with bright foci that colocalize with endosomes. Does NEKL-3 localize to the ER? The ER contacts many membrane-bound organelles and is implicated in fission of these compartments. The roles of NEKL-2 and NEKL-3 in membrane scission suggests that they could localize to ER::endosome contacts to regulate endosome fission. This would be an interesting avenue to pursue in the future.

We appreciate this suggestion. We had also previously noticed a variable/subtle cytoplasmic or reticulated pattern for NEKL-3 (and to a lesser extent NEKL-2), in our NEKL strains endogenously tagged with neonGreen and mKate (PMID: 27799278). To address this point, we generated a hyp7-specific ER marker (phyp7::vit-2Sig::oxGFP::KDEL) and crossed this into a CRISPR NEKL-3::mKate line. We've included an image to give a sense of the result (see below).

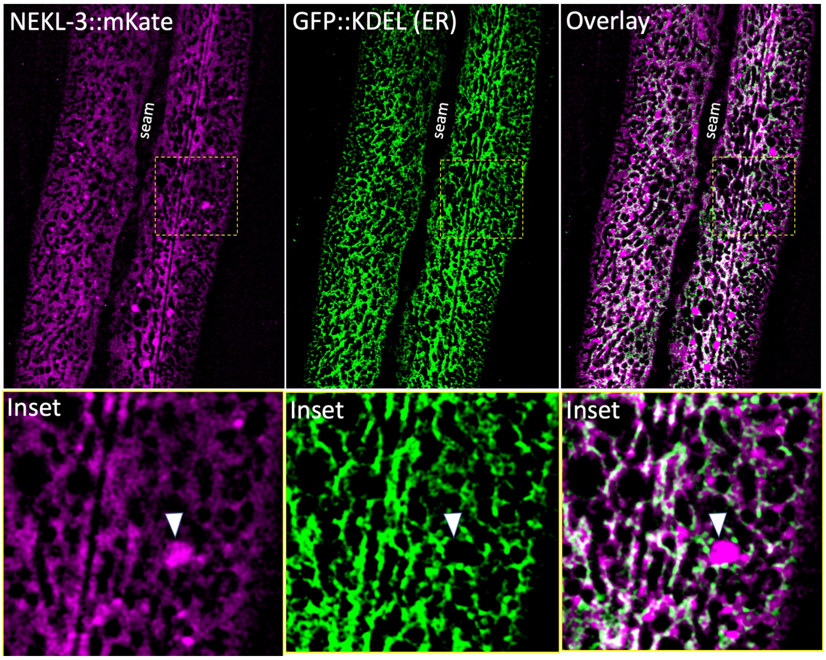

Although we do see some overlap, it's not clear how meaningful this is due to somewhat diffuse NEKL-3::mKate cytoplasmic localization along with the widespread GFP-ER signal. It is noteworthy that the (green) ER meshwork appears to encapsulate the bright (cyan) spherical NEKL-3 accumulations, which likely represent early and/or late endosomes. Since NEKL-2 also shows a similar background pattern, we do plan to cross the ER marker into the NEKL-2::mKate strain as well. Regardless, although this is a potentially interesting direction to pursue, we feel that this specific data doesn't coherently add to the current story, so we chose not to include it with this resubmission. Nevertheless, we now acknowledge the presence of some additional cytoplasmic localization NEKLs in the Results (lines 139–141).

We do, however, now show co-expression of a NEKL-3::mCherry reporter and AMAN-2::GFP (Golgi marker; Fig S3D). Lack of co-localization between these markers is consistent with an absence of clear changes in Golgi morphology observed in NEKL::AID depleted strains. These findings further support our statement in original manuscript that the reduction in Golgi vesicles observed in NEKL-3::AID depleted worms is likely to be caused by indirect effects (lines 204–207).

Mutation of cup-5 increases TGN-38 levels in NEKL-2/3 depleted animals. Does it also increase TGN-38 levels in a wild-type background? In other words, is it specific to the NEKL depleted animals?

We addressed this question by generating a strain containing TGN-38::GFP in a cup-5 (partial-LOF) mutant background. Our findings suggest that some portion of TGN-38 is normally delivered to the lysosome in wild type animals and that it's degradation depends on lysosomal function (i.e., cup-5), (Fig S5; lines 278–281).

The finding that TGN-38 is both recycled and degraded isn't surprising. What was somewhat unexpected was that the appearance of TGN-38 in cup-5 mutants differed qualitatively from the appearance of TGN-38 in the NEKL::AID; cup-5 strains. Namely, TGN-38 accumulations were larger in cup-5 single mutants and seem likely to represent enlarged (maturation defective) lysosomes. In contrast, TGN-38 foci appear consistently smaller in the NEKL::AID; cup-5 strains (Fig 4). In fact, we retested the NEKL::AID strains and again saw the same pattern shown in Fig 4. One possible explanation for this difference is that NEKL depletion may affect lysosomal morphology or lysosome abundance, which is something we plan to address in the future but now mention in the Results (lines 281–284).

In summary, the core observation that NEKL depletion leads to reduced TGN-38, which can be partially alleviated by inhibition of cup-5, suggests that TGN-38 may be abnormally directed to lysosomes for degradation in NEKL depleted worms.

Is kinase activity required for NEKL-2 and 3 function?

Yes. We have added this information to the Introduction along with relevant references (lines 87–89).

Figure 3. Is it possible to discern the basolateral membrane in wild-type animals as a point of reference using arrows? There is clearly an accumulation of SMA-6 and DAF-4, however the boundary is not clear in wild-type.

The reviewer is correct in that it is often not possible to discern the precise lateral and basolateral boundaries of hyp7 in the SMA-6::GFP and DAF-4::GFP lines in wild type. This is partly because of the curved rounded nature of the lateral membrane but also because of there are often very low levels of these cargos at the membrane. In theory, an additional marker that

is specific for the hyp7 plasma membrane could be generated for future use. We now briefly address this in the legend for Fig 3.

Reviewer #2:
This manuscript submitted to PLoS Genetics by Joseph et al. reports the functions of two conserved NIMA-related kinases, NEKL-2 (NEK8/9 homolog) and NEKL-3 (NEK6/7 homolog), in endocytic trafficking in both C. elegans and mammalian tissue culture cells. The Fay Lab previously reported that NEKL-2 and NEKL-3 are essential for C. elegans molting and that they regulate apical clathrin-mediated endocytosis in the epidermis. In this study, the authors found that NEKL-2 and NEKL-3 are differentially localized to, and regulate the morphology of, distinct endosomal compartments. They also provided evidence that NEKL-2 and NEKL-3 control the sorting of cargoes between different intracellular compartments, and the endocytosis of two BMP receptors from the basolateral surface. Furthermore, they showed that knocking down the NEKL-3 homologs in human cell lines also affects endosomal morphology, suggesting that the functions of these NIMA related kinases in regulating endocytic trafficking is conserved.

The manuscript is very well written. It has identified new roles for members of the NIMA family of kinases, which are important in human development and disease. I have one main concern. The authors showed that depletion of NEKL-2 or NEKL-3 affects the uptake of both SMA-6, a clathrin-dependent cargo, and DAF-4, a clathrin-independent cargo, from the basolateral surface of epidermal cells. The authors speculated that these two receptors may be trapped in early endosomes near the basolateral surface. In light of their previous findings that NEKL-2 and NEKL-3 regulate apical clathrin-mediated endocytosis in the epidermis, it will be important to determine whether additional clathrin-independent cargoes are also affected upon NEKL-2 or NEKL-3 depletion.

We appreciate the reviewer’s point about clathrin-dependent versus independent roles for NEKLs in endocytosis. We tried to address this by examining a model cargo, hTAC::GFP, which exhibits uptake by a clathrin-independent mechanism in some other systems and cell types including the C. elegans intestine. However, we encountered several issues. One is that, based on an apa-2 mutant that we tested, hTAC::GFP may exhibit partial dependence on AP2/clathrin for uptake in the epidermis, thus ruling it out as a clear readout for clathrin-independent endocytosis. This is perhaps not entirely surprising as hTAC uptake has also been reported to partially depend on CME in some contexts (PMID: 29549600). Moreover, although we observed strong effects on the subcellular localization of hTAC::GFP in NEKL-2::AID and NEKL-3::AID strains, it appeared that this cargo was being retained primarily in enlarged internal vesicles of unknown identify. Given the lack of a clear result–interpretation for hTAC, we have not included this data in our revision.

The above discussion also has bearing on our investigation of the basolateral cargos SMA-6 (type I TGF-Beta Receptor – AP2/CME dependent) and DAF-4 type II TGF-Beta Receptor – AP2/CME independent). At the time of submission we had not been able to construct a NEKL-2::AID strain in which to test DAF-4. More recently we were able to generate this strain and found that loss of NEKL-2 had no significant effect on DAF-4::GFP localization (Fig 3, lines 234–

242), indicating that NEKL-2 is not required for the uptake of this cargo. Although this could suggest that NEKL-2 is specifically required for CME, this interpretation must be tempered because the effect of NEKL-2 loss on SMA-6 was relatively weak as compared with NEKL-3, which strongly affected both SMA-6 and DAF-4. Thus, NEKL-2 may simply play a more minor role at the basolateral membrane, at least with respect to the TGF-beta receptors.

Overall, our findings indicate that NEKL-3 can affect both clathrin-dependent (SMA-6) and clathrin-independent (DAF-4) cargos at the basolateral membrane. One potential caveat, however, is that in the presence of ligand, SMA-6 and DAF-4 would be expected to form a complex, and thus SMA-6 or DAF-4 could potentially affect each other's uptake or retention. For example, membrane retention of endogenous SMA-6 could potentially impact the localization and uptake of DAF-4::GFP in NEKL-3 depleted worms, a point that we bring up in the Discussion section (lines 384–392). Nevertheless, based on PMID: 31988138 and PMID:11784045, endogenous SMA-6 is expressed at very low levels (e.g., CRISPR'd SMA-6::GFP is undetectable by fluorescence microscopy), and thus it seems unlikely that endogenous plasma-membrane associated SMA-6 would indirectly lead to strong membrane-juxtamembrane retention of highly expressed DAF-4::GFP in the NEKL-3::AID strains. Altogether, these findings do suggest roles for NEKL-3 in both CME and non-CME.

Furthermore, the data (at least those presented in Figure 2) do not seem to show accumulation of early endosomes upon NEKL-2 or NEKL-3 depletion. Maybe the accumulation of SMA-6 and DAF-4 is not due to defects in intracellular trafficking upon NEKL-2 and NEKL-3 depletion, rather, due to some other un-identified role(s) of NEKL-2 and NEKL-3 on the cell surface.

The reviewer raises a good point in that this finding suggests that the accumulation of SMA-6 or DAF-4 at or near the BL membrane is likely to be on the membrane or at least in a juxtamembrane vesicle or compartment that is not marked by RAB-5 and thus likely not early endosomal in identify. We have added this point to the Discussion (lines 396–402).

I have several additional comments listed below:
1) I wonder if Figure 1 is somehow mis-labeled. The images and the quantifications do not seem to match. The authors concluded, based on the quantification shown in panel M, that NEKL-2 has substantial co-localization with the early endosome marker RAB-5, but not with the late endosome marker RAB-7, while NEKL-3 has substantial co-localization with both RAB-5 and RAB-7. However, based on the images shown in Figure 1, NEKL-2 (but not NEKL-3) seems to have substantial co-localization with both RAB-5 and RAB-7 (1I' and 1L'), while NEKL-3 appears to primarily co-localize with RAB-7 (1C' and 1F').

This was a good catch and we have replaced the aforementioned panels (Fig 1J–L) with ones that are more representative of the typical overlap that we observe. Still, we do indeed see some worm-to-worm variability in overlap (e.g., ~2–25% for NEKL-2 with RAB-7), as reflected by the Manders' plot (Fig 1M).

2) The NEKL-2::mNeonGreen and NEKL-3::mNeonGreen signals shown in Figure S1 appear to be

significantly brighter than those shown in Figure S2. Is this due to differences in different focal planes imaged or to other reasons?

The reviewer is correct. This data (was collected on a different confocal scope as indicated in original materials and methods and now mentioned in the Fig S2 legend.

3) In Figure 3, the diameters of NEKL-3::AID worms (panels E and G) seem to be much smaller than those of WT or NEKL-2::AID worms (panels C, D and F). Is this difference due to differences in focal planes and/or stages of the animals examined, or due to NEKL-3::AID worms being unhealthy after NEKL-3 depletion?

NEKL-3::AID worms are indeed sicker and smaller relative to wild type even in the absence of auxin. This may be attributable to a partial LOF of NEKL-3 caused by the AID tag (Joseph et al., 2020). We now mention this in the text (lines 189–192).

4) Based on the western blots shown for both NEK6 and NEK7 in Figures 5-7, there seems to be substantial variation in the efficiency of the siRNA knockdown experiments. Is there any correlation between the knockdown efficiency and the severity of the phenotypes observed? Along the same line, the antibodies for NEK7 used for the western blots shown in Figures 5-7 seem questionable. It is unclear which band is specifically NEK7. In Figure 5, NEK7 appears to be the bottom one of two bands, while in Figure 6, it appears to be the middle one of three. Surprisingly, the intensities of the bottom two bands are both drastically reduced in Figure 7. The authors may want to either repeat the western blots, or comment on the specificity of the antibody.

We thank the reviewer for these thoughtful comments. Regarding siRNA knock-down efficiency, there is indeed some biological viability between experiments. However, all experiments did yield an approximate efficiency range between 65-90% knock-down, which is fairly typical for mammalian cells. Unfortunately, it is not possible to correlate efficiency with the phenotypes observed in individual cells, since the efficiency is measured per cell population as a mean, whereas we have no way to know the levels of knock-down in individual cells.

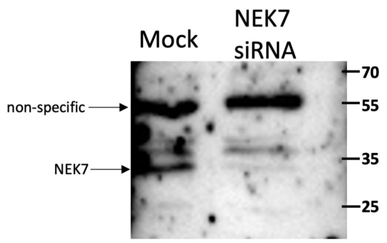

Regarding the anti-NEK7 antibody used: there are indeed some differences in the background bands that are observed between individual experiments. While we cannot account for these differences, the validity of the NEK7 band is inferred by its correct molecular weight, as well as (significantly) this band being the one that is reduced or lost upon siRNA knock-down. Nonetheless, we have run an additional immunoblot analysis on mock- and knockdown cells and provide the data here. Again, we do observe a specific reduction in the band corresponding in size to NEK7.
